# The small non-coding RNA B11 regulates multiple facets of *Mycobacterium abscessus* virulence

**DOI:** 10.1371/journal.ppat.1011575

**Published:** 2023-08-21

**Authors:** Michal Bar-Oz, Maria Carla Martini, Maria Natalia Alonso, Michal Meir, Nicola Ivan Lore, Paolo Miotto, Camilla Riva, Shiva K Angala, Junpei Xiao, Catherine S Masiello, Maria-Anna Misiakou, Huaming Sun, Justin K Moy, Mary Jackson, Helle Krogh Johansen, Daniela Maria Cirillo, Scarlet S Shell, Daniel Barkan

**Affiliations:** 1 Koret School of Veterinary Medicine, Faculty of Agriculture, Food and Environment, The Hebrew University of Jerusalem, Rehovot, Israel; 2 Worcester Polytechnic Institute, Worcester, Massachusetts, United States of America; 3 Rambam Medical Centre, Haifa, Israel; 4 IRCCS San Raffaele Scientific Institute, Milan, Italy; 5 Mycobacteria Research Laboratories, Department of Microbiology, Immunology and Pathology, Colorado State University, Fort Collins, Colorado, United States of America; 6 Center for Genomic Medicine, Copenhagen University Hospital-Rigshospitalet, Copenhagen, Denmark; 7 Department of Clinical Microbiology, Rigshospitalet, Copenhagen, Denmark; McGill University, CANADA

## Abstract

*Mycobacterium abscessus* causes severe disease in patients with cystic fibrosis. Little is known in *M*. *abscessus* about the roles of small regulatory RNAs (sRNA) in gene regulation. We show that the sRNA B11 controls gene expression and virulence-associated phenotypes in this pathogen. B11 deletion from the smooth strain ATCC_19977 produced a rough strain, increased pro-inflammatory signaling and virulence in multiple infection models, and increased resistance to antibiotics. Examination of clinical isolate cohorts identified isolates with B11 mutations or reduced expression. We used RNAseq and proteomics to investigate the effects of B11 on gene expression and test the impact of mutations found in clinical isolates. Over 200 genes were differentially expressed in the deletion mutant. Strains with the clinical B11 mutations showed expression trends similar to the deletion mutant, suggesting partial loss of function. Among genes upregulated in the B11 mutant, there was a strong enrichment for genes with B11-complementary sequences in their predicted ribosome binding sites (RBS), consistent with B11 functioning as a negative regulator that represses translation via base-pairing to RBSs. Comparing the proteomes similarly revealed that upregulated proteins were strongly enriched for B11-complementary sequences. Intriguingly, genes upregulated in the absence of B11 included components of the ESX-4 secretion system, critical for *M*. *abscessus* virulence. Many of these genes had B11-complementary sequences at their RBSs, which we show is sufficient to mediate repression by B11 through direct binding. Altogether, our data show that B11 acts as a direct negative regulator and mediates (likely indirect) positive regulation with pleiotropic effects on gene expression and clinically important phenotypes in *M*. *abscessus*. The presence of hypomorphic B11 mutations in clinical strains is consistent with the idea that lower B11 activity may be advantageous for *M*. *abscessus* in some clinical contexts. This is the first report on an sRNA role in *M*. *abscessus*.

## Author summary

*Mycobacterium abscessus* is a bacterial pathogen that causes difficult-to-treat disease. To develop better treatments for *M*. *abscessus* infections, we need to better understand how it adapts to the stresses imposed by antibiotics and the immune system. Bacteria, and specifically pathogens, need to regulate gene expression to control their physiology and virulence. In better studied bacteria, small non-coding RNAs (sRNAs) have been shown to participate in gene regulation. In contrast, almost nothing is known about the roles of sRNAs in *M*. *abscessus*. Here, we identified an sRNA in *M*. *abscessus* called B11 (also present in other mycobacterial pathogens) that is a pleotropic regulator of multiple pathways–both increasing and decreasing the expression of ~200 genes belonging to pivotal pathogenesis phenotypes. Deletion of B11 resulted in a hyper-inflammatory, hyper virulent strain, via altered expression of genes in multiple virulence pathways. We experimentally show that for some genes, the mechanistic basis for this regulation is direct binding of the sRNA to target mRNA via specific basepairing. It appears that deletion of B11 increases the virulence of the bacterium–and this notion is supported by the finding of B11 hypomorphic mutations in three unrelated cohorts of clinical isolates of *M*. *abscessus*.

## Introduction

Non-tuberculous mycobacteria are increasingly recognized as human pathogens [1–4]. Of these, *Mycobacterium abscessus* is associated with the most severe chronic infections, including virtually incurable pulmonary disease in patients with cystic fibrosis (CF) [5–7]. People with diabetes mellitus, kidney disease, malignancies, COPD, and compromised immune systems also appear to have increased susceptibility to *M*. *abscessus* infection and disease (8–10[8–10]). Bacterial virulence requires regulation of gene expression to facilitate phenotypic adaptation to various niches within the human host. However, knowledge of the fundamental biology underlying gene regulation and phenotypic adaptations in *M*. *abscessus* lags far behind that of *M*. *tuberculosis* (Mtb), in part due to lack of genetic tools and in part due to the relatively recent recognition of *M*. *abscessus* as an important human pathogen.

Colony morphology is often considered a correlate of *M*. *abscessus* virulence. Patients are typically infected with smooth (S) variants. The S morphotype is conferred by the presence of glycopeptidolipids (GPL) in the cell envelope. Rough (R) variants, not producing GPL, typically arise later in infection [11,12] and may be associated with worse outcomes[13]. S and R morphotypes exhibit different growth characteristics in macrophages [14], and different stimulation of innate immunity [15–18]. Overall, S morphotypes are typically associated with intracellular growth and blockage of phagolysosomal fusion while R morphotypes are associated with extracellular growth and pro-inflammatory signaling that causes severe tissue destruction [12,14,19–21]. However, opposite effects have been shown with respect to intracellular growth highlighting the complexity of this phenotype[15]. The switch is usually due to mutations in *gpl* biosynthesis genes such as mps1 and mps2 (*MAB_4099c*, *MAB_4098c*, respectively) or GPL transporter genes such as *mmpL4b (MAB_4115c)* [22,23].

As in Mtb, ESX secretion systems are present in *M*. *abscessus* and involved in virulence. The ESX-4 system was shown to be important for survival of *M*. *abscessus* in macrophages and may play a role in phagosomal permeabilization, functionally analogous to the role of ESX-1 in Mtb [24]. However, little is known about how expression and function of the ESX-4 system and other *M*. *abscessus* virulence factors are regulated.

Small non-coding RNAs (sRNAs) contribute to pathogenesis and regulation of virulence in bacteria including *Listeria monocytogenes* [25], *Staphylococcus aureus* [26], and *Salmonella enterica* [27,28]. sRNAs have been identified in mycobacteria, mostly by RNAseq screens [29–32]. Some are expressed in specific conditions, giving clues regarding their roles [33]. The *M*. *smegmatis* sRNA Ms1 is upregulated in stationary phase and interacts with core RNA polymerase, repressing transcription by preventing holoenzyme formation [34]. Consistently, the Mtb homolog MTS2823 is highly expressed in stationary phase, and ectopic overexpression in log phase caused downregulation of energy metabolism genes [30]. Mcr11 is involved in regulation of fatty acid metabolism in Mtb [35], whereas Mcr7 is involved in TAT secretion [36]. MrsI is induced by multiple infection-associated stressors and required for mycobacterial growth in low-iron conditions [37]. MTS1338 was suggested to trigger dormancy-like gene expression changes in response to infection-associated conditions in Mtb [38] as well as to mediate response to oxidative stress [39]. Orthologs of some of these sRNAs were identified in a transcriptomics study of *M*. *abscessus* [40]. However, almost nothing is known about roles played by sRNAs in this important emerging pathogen.

The sRNA B11 was identified in Mtb and has also been studied in *M*. *smegmatis* (reviewed in [41]). It has a conserved secondary structure in many mycobacteria with two unstructured loops containing strings of cytosines. Overexpression of Mtb B11 in *M*. *smegmatis* was reported to cause slow growth and repress several genes, due in some cases to base-pairing of B11’s cytosine-rich loops to genes’ ribosome binding sites (RBSs) and presumably competing with ribosomes [29,42]. Overexpression of B11 in Mtb fully suppressed growth [29]. Also in Mtb, B11 had reduced expression in stationary phase and a modest increase of expression in response to oxidative stress [29]. Disruption of B11 by a transposon in *Mycobacterium kansasii* caused a growth defect and a conversion from rough to semi-smooth colony morphology [43].

Here we screened an *M*. *abscessus* transposon mutant library and identified the sRNA B11 as a factor required for smooth morphology. We constructed a targeted B11 deletion mutant and characterized it for gene expression and pathogenesis-associated phenotypes. We found that loss of B11 caused increased virulence and pro-inflammatory immune signaling as well as altered expression of a large set of genes enriched for those with B11-complementary sequences in their RBSs. Genes with increased expression in the B11 deletion mutant included several components of the ESX-4 secretion system, at least one of which is repressed by direct binding of B11 to its RBS. Furthermore, B11 is mutated in several clinical isolates. Strains with these mutations have gene expression patterns consistent with reduced B11 activity, suggesting that reduced B11 function is advantageous to the bacteria in some clinical contexts.

## Results

### Loss of the sRNA B11 causes rough colony morphology in *M*. *abscessus*

**Fig 1:**
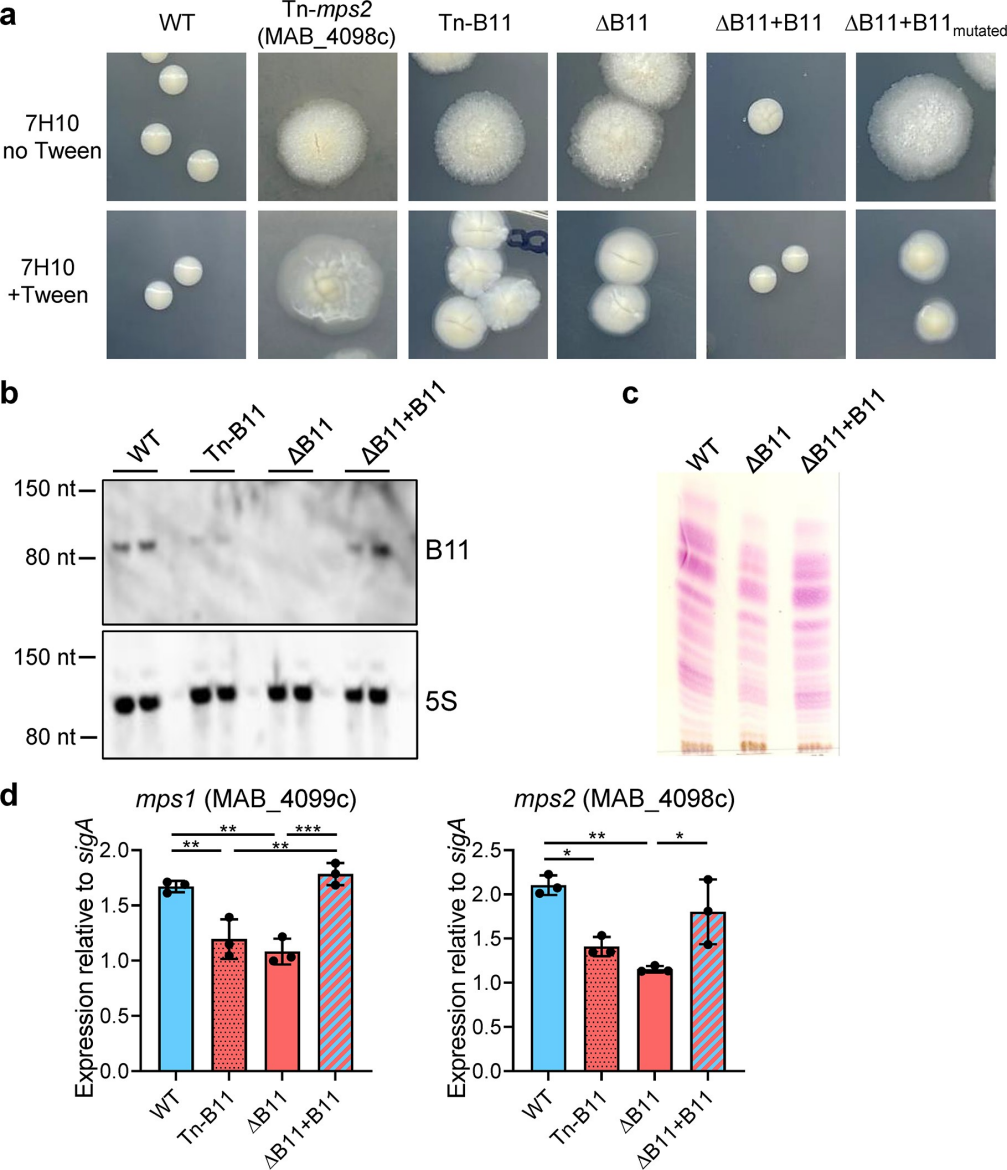
Disruption of the sRNA B11 causes rough morphology with reduced GPL content in *M*. *abscessus*. ** A.** The indicated strains were grown in liquid media with Tween 80, diluted and plated on solid media with and without Tween 80. Colonies were imaged after 7 days of growth. **See S**1**E Fig for exact mutations of ∆B11+B11**_**mutated.**_ **B.** Northern blotting of duplicate RNA samples from the indicated strains confirmed that B11 expression was reduced in the transposon mutant, absent from the deletion mutant, and restored in the complemented mutant. **C.** Thin layer chromatography analysis of surface lipids extracted from the indicated strains grown on 7H10-ADC agar plates. Lipids extracted from equivalent amounts of bacterial cells were analyzed on silica gel 60-precoated TLC plates F254 (Merck) developed in the solvent system chloroform:methanol:water (90:10:1, v/v/v). The plate was revealed by spraying with α-naphthol and heating. **D.** Quantitative PCR reveals that expression of two key GPL biosynthesis genes is modestly but significantly reduced in strains lacking B11. * *P* < 0.05; ** *P* < 0.01; *** *P* < 0.001; ANOVA with Tukey post-test. qPCR comparisons where *P* > 0.05 are not shown. qPCR data represent triplicate log-phase cultures. All experiments shown in this figure were performed at least twice and representative data are shown.

While mutations in GPL biosynthesis and transport genes are known to cause conversion of smooth *M*. *abscessus* to rough, we sought to determine if there were additional genetic paths to the rough morphotype. We therefore constructed a transposon mutant library in *M*. *abscessus* ATCC_19977 (S morphotype) [44] and screened it for rough colonies. Some rough colonies had transposons inserted in GPL biosynthesis and transport genes (*mps1*, *mps2*, and *mmpL4b*), as expected [22]. We also identified a rough mutant with a transposon inserted in the -10 sigma factor binding site of the promoter for the sRNA B11 (Figs 1A, S1A and S1B). The Tn mutant had substantially less B11 transcript than the parental strain (Fig 1B, WT and Tn-B11). Mycobacterial promoters typically contain the core -10 sequence TANNNT [45–47]. As the Tn left this core sequence intact, B11 was likely expressed from a fusion of its native promoter and the 3’ portion of the Tn.

To determine if reduced B11 expression was responsible for the rough morphology of the Tn mutant, we replaced the native B11 gene in ATCC_19977 with a *zeocin*^*R*^ gene, creating a complete B11-deletion mutant. The ΔB11 mutant had characteristic rough morphology and exhibited extensive clumping in liquid culture, especially in media without Tween (Figs 1A, S1C and S1D). Both phenotypes reverted to WT in a complemented mutant with one copy of B11 transcribed from the native promoter, integrated at the L5 site (ΔB11+B11) (Fig 1A and 1B). However, the colony morphology was not complemented by a version of B11 with multiple mutations in one of the unstructured loops (Fig 1A rightmost panel and S1E Fig for details of the mutations), implicating the loop as playing a functionally important role.

To investigate the mechanistic basis of the rough morphology of ΔB11, we first examined the GPL content in lipids extracted from bacterial membranes, using TLC. Although the ΔB11 mutant still produced GPL, the amount was reduced as compared to WT, and returned to WT levels upon complementation with a functional B11 gene (Fig 1C). LC-MS of extracted cell envelope lipids indicated that the relative abundance of diglycosylated and triglycosylated GPLs with different carbon chain lengths were similar in the presence and absence of B11 (S2 Fig.), indicating that loss of B11 leads to quantitative changes in GPL abundance but not qualitative changes in the types of GPL present. We then measured expression of two key GPL biosynthesis genes, *mps1* (*MAB_0499c*) and *mps2* (*MAB_0498c*). Both had significantly reduced expression in the ΔB11 mutant and returned to WT levels upon complementation (Fig 1D), suggesting the rough morphology was due in part to reduced expression of GPL biosynthesis genes.

### A B11 deletion strain has virulence and envelope-leakiness properties reminiscent of “classic”, GPL-related rough strains

**Fig 2:**
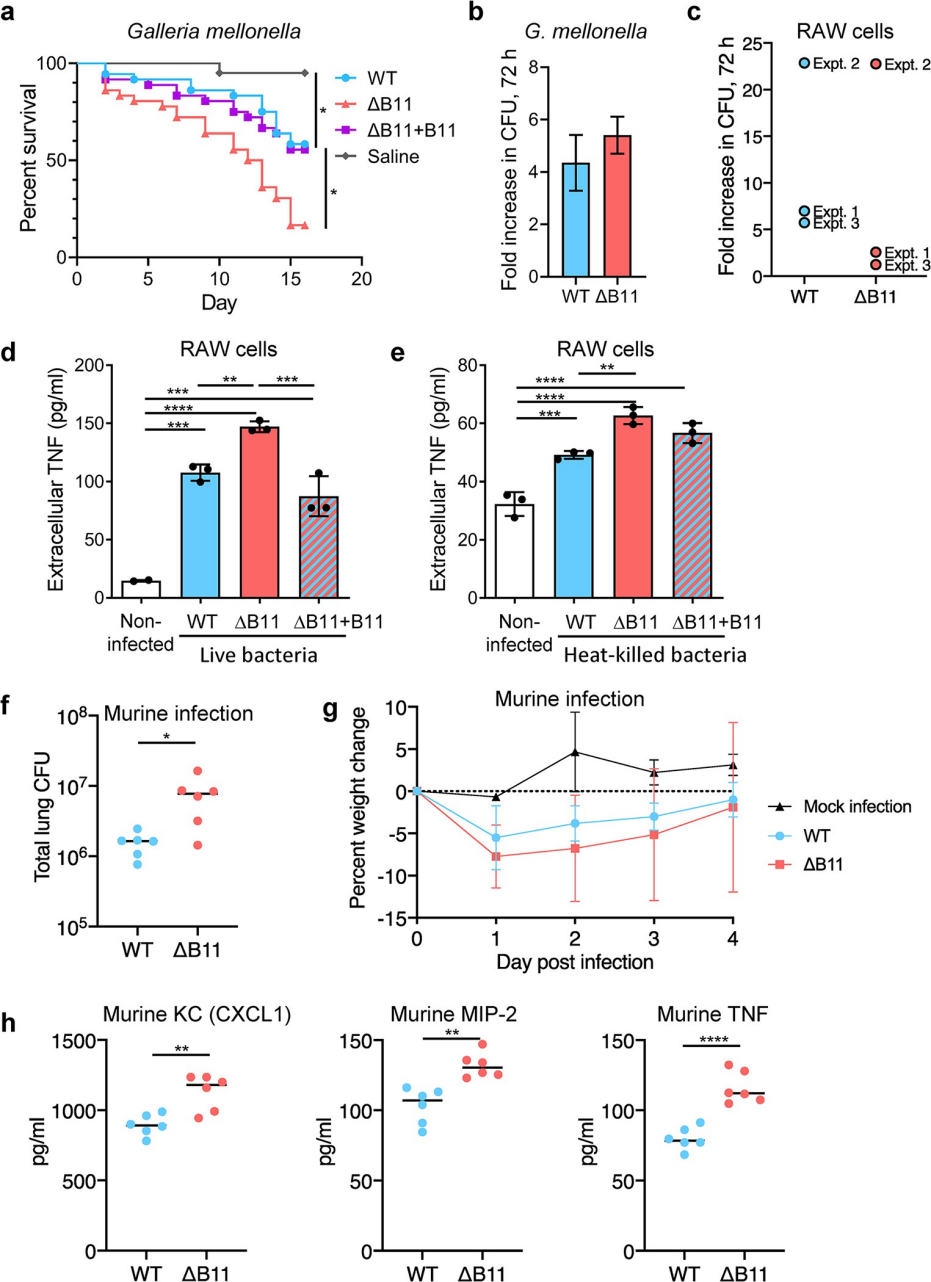
Disruption of *M*. *abscessus* B11 increases virulence and cytokine secretion. **A.** *Galleria mellonella* infections. 36 larvae per group were infected by ~3000 CFU of WT *M*. *abscessus*, the ΔB11 mutant, or the ΔB11+B11 complemented strain. 20 larvae were injected with saline. Larvae were followed for two weeks. The survival in each group is showed in a Kaplan-Mayer curve. The survival of infection with WT *M*. *abscessus* was compared to each other group by log-rank (Mantel-Cox) test. *, *P* < the Bonferroni-corrected threshold of 0.0167. **B.** 20 larvae were infected by WT, and 20 by the ΔB11 mutant, ~1000 cfu/larva. 10 larvae from each group were sacrificed on day 0, and the remaining on day 3, and CFU were enumerated by plating. Y axis represents proliferation at day 3 over day 0, showing no significant advantage of either strain. **C.** RAW macrophage-like cells were infected with the indicated *M*. *abscessus* strains and bacteria were enumerated three days later. Three separate experiments were performed (expt. 1-expt. 3) with 2–4 replicates per experiment. The mean fold-increase for each experiment is shown. **D.** RAW cells were infected with *M*. *abscessus* at an MOI of 5 and TNF secretion was measured after 6 hours. **E.** RAW cells were exposed to heat-killed *M*. *abscessus* at the equivalent to an MOI of 20, washed, and TNF secretion measured after 24 hours. **F.** Lung bacterial burden for C57BL/6NCrl mice four days after infection with the indicated *M*. *abscessus* strains. **G.** Weight change in mice following infection with the indicated *M*. *abscessus* strains or mock infection with PBS-infused agar beads. Points are error bars represent mean and SD. n = 9 mice per group for infections and 3 mice for the mock infection. **H.** The indicated cytokines were measured in lung homogenates four days after infection with the indicated *M*. *abscessus* strains. Bar charts display means and SD. Black dots indicate individual samples. ** *P* < 0.01; *** *P* < 0.001; **** *P* < 0.0001, ANOVA with Tukey post-test. All experiments shown in this figure except for the murine infections were done at least twice and representative data are shown.

Rough *M*. *abscessus* strains have been reported to be more virulent and more pro-inflammatory. We therefore wondered if the ΔB11 mutant differed from its smooth WT parental strain with respect to these properties. To assess virulence, we first used our previously established *Galleria mellonella* infection model [48]. We infected larvae with WT *M*. *abscessus* (ATCC_19977), the ΔB11 mutant, a complemented mutant (ΔB11+B11), and a saline control (36 larvae/group, 20 in the saline control). Equal inocula were confirmed by immediate plating of an additional three homogenized larvae from each group. Larval survival was significantly reduced in the ΔB11-infected group (Fig 2A), and returned to WT levels upon complementation. Hypervirulence is often associated with significantly higher bacterial proliferation, as measured by CFU. However, a separate experiment showed bacterial proliferation did not significantly differ in larvae infected by the ΔB11 mutant as compared to WT (Fig 2B).

To assess the effects of B11 on intracellular replication and innate immune activation, we infected RAW mouse macrophage-like cells with ATCC_19977 or the ΔB11 mutant. Proliferation of the ΔB11 mutant after 72 hours was comparable to that of WT (Fig 2C). However, RAW cells infected with the ΔB11 mutant secreted more TNF than those infected by the WT or complemented strains (Fig 2D), suggesting stronger activation of an inflammatory response [49]. RAW cells exposed to heat-killed ΔB11 bacteria also secreted more TNF than those exposed to heat-killed WT bacteria (Fig 2E), indicating that this response may result directly from the modified cell envelope of the deletion mutant.

To determine if the hyper-inflammatory phenotype was preserved in a whole animal experimental model, we infected mice with the WT and ΔB11 strains, using our established agar-beads infection model [50,51]. Bacterial burden after four days of infection was significantly higher for mice infected with ΔB11 compared to those infected with wildtype (Fig 2F). Similarly, mice infected with ΔB11 exhibited a trend towards more weight loss (Fig 2G) and had substantially higher lung levels of the pro-inflammatory cytokines TNF, Keratinocyte-derived Cytokine (KC, also known as CXCL1), and Macrophage-Inflammatory Protein-2 (MIP-2, also known as CXCL2) (Fig 2H).

To further compare the cell-envelope-related properties of the ΔB11 strain, we examined the proteins released into culture supernatants. The ΔB11 strain released substantially more proteins of various sizes compared to the WT and complemented strains (S3A Fig). A “classic” rough strain in which the GPL biosynthesis gene *mps2* was disrupted by a transposon (Tn-*mps2*) similarly released more proteins into the culture supernatant, suggesting this may be a general property of rough strains (S3B Fig). To determine if the greater protein abundance in the culture supernatants was due exclusively to increased secretion, we transformed the strains with a plasmid encoding cytoplasmic mCherry and probed the culture supernatants and cell lysates by western blot. In the WT strain mCherry was detected only in cell lysates, while in the ΔB11 and Tn-*mps2* strains it was detected in the culture supernatants as well (S3C Fig). As mCherry does not contain a secretion signal sequence, this result suggests that rough strains may be generally leakier, with a greater amount of cytoplasmic protein being released during normal growth.

### B11 affects drug resistance

The ΔB11 mutant was also more resistant to two antimycobacterial drugs. The MICs of linezolid and rifampicin were elevated 4-fold and 8-fold, respectively. In contrast, only a trivial difference was found in the MIC of ciprofloxacin (Table 1), and no change was found in the MICs of meropenem, amikacin, azithromycin, or tigecycline. To our knowledge rough morphology *per se* has not been reported to affect drug resistance, specifically not at these concentrations [52]; these effects may therefore be due to other physiological changes in the ΔB11 mutant.

**Table 1:** *M*. *abscessus* ∆B11 is more resistant to linezolid and rifampicin as compared to its WT and complemented (∆B11+B11) counterparts^a^.

		WT *M*. *abscessus*^*Lux*^ *(ATCC 19977)*	*M*. *abscessus*^*Lux*^ ∆B11	*M*. *abscessus*^*Lux*^ ∆B11+B11
**Ciprofloxacin**	MIC_90_	0.3 μg/ml	0.3 μg/ml	0.3 μg/ml
	MIC_99_	0.6 μg/ml	0.6 μg/ml	0.6 μg/ml
**Linezolid**	MIC_90_	0.8 μg/ml	1.6 μg/ml	0.8 μg/ml
	MIC_99_	1.6 μg/ml	12.5 μg/ml	1.6 μg/ml
**Rifampicin**	MIC_90_	2.35 μg/ml	4.7 μg/ml	4.7 μg/ml
	MIC_99_	4.7 μg/ml	37.5 μg/ml	9.4 μg/ml

^a^All drugs were tested in 2-fold dilutions in triplicate wells. For each strain/drug combination, the drug concentrations corresponding to 90% and 99% growth inhibition were the same across the triplicate wells.

### B11 affects expression of a large gene set enriched for genes with B11-complementary sequences in their 5’ UTRs

**Fig 3:**
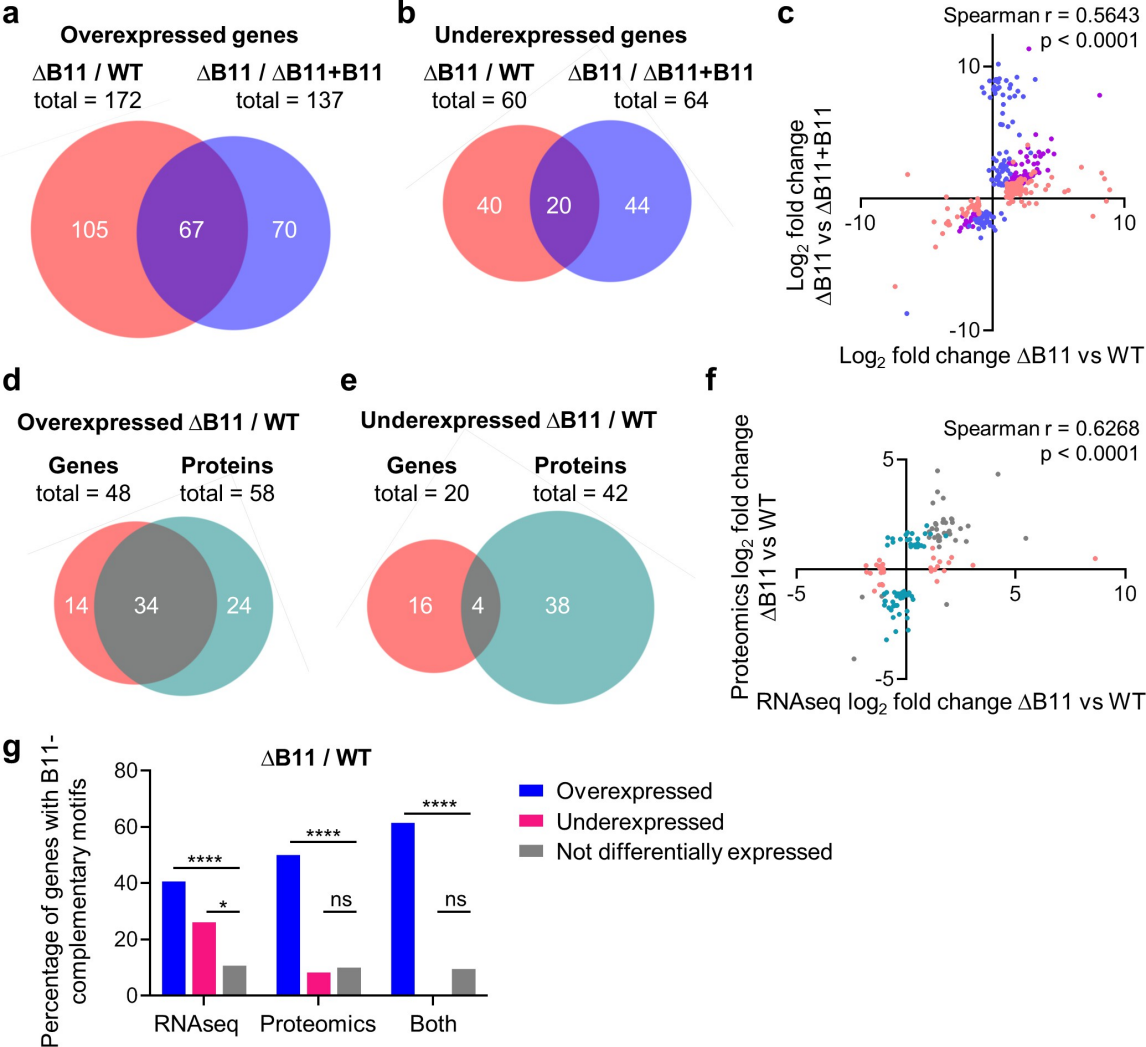
Disruption of the sRNA B11 in *M*. *abscessus* causes gene expression changes at the mRNA and protein levels particularly in genes with B11-complementary ribosome-binding sites. **A-C.** DESeq2 (93) was used to identify genes differentially expressed in a ∆B11 strain compared to a WT strain and in a ∆B11 strain compared to a∆B11 complemented with B11 expressed from an ectopic location (∆B11+B11). mRNA abundance was measured by RNAseq. Genes that met the thresholds of > = 2-fold mRNA abundance change and adjusted *P* < 0.05 were classified as differentially expressed. **A-B,** Venn diagrams show genes that met the criteria for overexpression or underexpression in the ∆B11 strain compared to the WT or complemented strains. Only genes with data in both datasets are shown. **C.** The log_2_ fold changes of genes that met the differential expression criteria for either comparison were plotted to assess their correlation. Pink dots indicate genes that met the differential expression criteria for the ∆B11/WT comparison only, blue dots indicate genes that met the differential expression criteria for the ∆B11/∆B11+B11 comparison only, and purple dots indicate genes that met the criteria for both comparisons. **D-F.** LC-MS/MS was used to compare protein abundance in the WT and ∆B11 strains. Proteins with > = 2-fold abundance differences and between-replicate CVs < 0.5 were classified as differentially expressed. **D-E,** Venn diagram show the overlap between genes that met the criteria for overexpression or underexpression by RNAseq (as in A-B) and the proteins that met the criteria for overexpression or underexpression by LC-MS/MS. Only genes/proteins with data in both datasets are shown. **F.** The log_2_ fold changes of genes that met the differential expression criteria for RNAseq or LC-MS/MS were plotted to assess their correlation. Pink dots indicate genes that met the differential expression criteria by RNAseq only, teal dots indicate genes that met the differential expression criteria by LC-MS/MS only, and gray dots indicate genes that met the criteria for both methods. **G.** Differentially expressed genes are enriched for the presence of B11-complementary sequences in their ribosome bind sites. Genes/proteins were categorized according to whether or not they met the differential expression criteria used in D-F, and the proportion of genes/proteins in each category that had > = 6 nt of consecutive sequence in the 25 nt upstream of their start codons that was complementary to one or both loops of B11 was determined. Only genes (and their encoded proteins) with defined transcription start sites were included in the analysis. * *P* < 0.05; **** *P* < 0.0001, Fisher’s exact test. All Venn diagrams were made by BioVenn (101).

Heterologous overexpression of *M*. *tuberculosis* B11 in *M*. *smegmatis* was previously shown to affect gene expression, in some cases through direct binding to RBSs [42]. To determine the effect of B11 on gene expression in *M*. *abscessus*, we performed whole-proteome analysis and RNAseq (S1 Table). RNAseq with the WT, ΔB11, and ΔB11+B11 strains revealed 254 genes that were differentially expressed in the deletion strain compared to the WT strain and 201 that were differentially expressed in the deletion strain compared to the complemented strain. Our criteria for differential expression were fold-change > = 2, adjusted *p* < 0.05. Over twice as many genes were overexpressed than underexpressed in the ΔB11 strain, consistent with previous postulation that B11 is a negative regulator in some mycobacteria [42]. There was substantial but imperfect overlap in genes that met our criteria for differential expression in the ΔB11 strain vs WT and ΔB11 vs ΔB11+B11 (Fig 3A–3B; *p* < 0.001, hypergeometric test). To better understand the behavior of genes that met the differential expression criteria for one comparison but not the other, we examined the correlation in log_2_ fold change values between genes differentially expressed in either or both comparisons (Fig 3C). Most genes had similar expression trends in both comparisons even when they only met the criteria for differential expression in one comparison. Genes that had discrepant expression trends included those adjacent to the B11 chromosomal locus (probably related to the constitutive promotor in the antibiotic resistance cassette). Additionally, a mercury-resistance plasmid naturally present in ATCC_19977 was apparently lost in the process of constructing the ΔB11 strain.

LC-MS/MS of whole cell lysates from WT and ΔB11 strains revealed 90 differentially abundant proteins (> = 2-fold expression change relative to WT and CVs <0.5 between replicates for each strain). As in the RNAseq experiment, more proteins were overexpressed than underexpressed in the ΔB11 strain Among the genes and proteins with high-confidence data in both the RNAseq and proteomics datasets, there was high overlap among the overexpressed genes/proteins and modest but still statistically significant overlap among underexpressed genes/proteins (Fig 3D–3E; *p* < 0.0001 for overexpressed genes/proteins and *p* = 0.0036 for underexpressed genes/proteins, hypergeometric test). When the correlations in log_2_ fold change were assessed, we found that a majority of genes/proteins shared the same expression trends even when not meeting the criteria for differential expression in both datasets (Fig 3F).

To investigate the possibility that some of the expression changes were due to direct base-pairing of B11 to RBSs, we quantified the presence of B11-complementary sequences in the 25 nt upstream of the start codons of genes with defined 5’ UTRs [40] (S1 Table). Among the genes and proteins that were overexpressed in the ΔB11 strain, 40–60% of those with defined 5’ UTRs had B11-complementary sequences of 6 nt or greater, compared to ~10% of non-differentially-expressed genes/proteins (Fig 3G). This represented a substantial and highly statistically significant enrichment for B11-complementary RBS sequences among the overexpressed genes and proteins. In contrast, only 26% of underexpressed genes and 8% of underexpressed proteins had B11-complementary sequences. As discussed in more detail in the discussion section, these results are consistent with the ideas that (i) B11 may directly repress translation [42] and (ii) reduced translation or ribosome binding frequently lead to mRNA degradation and/or premature transcriptional termination in bacteria ([53–59] and reviewed in [60]). Genes affected by B11 deletion that do not have B11-complementary regions in their 5’ UTRs may be affected indirectly by transcription factors that are in turn directly or indirectly affected by B11. The finding that underexpressed genes also had a modest enrichment for B11-complementary sequences suggests that B11 may in some cases also stabilize transcripts by binding to their RBS regions.

### B11 represses expression of several ESX-related genes

**Fig 4:**
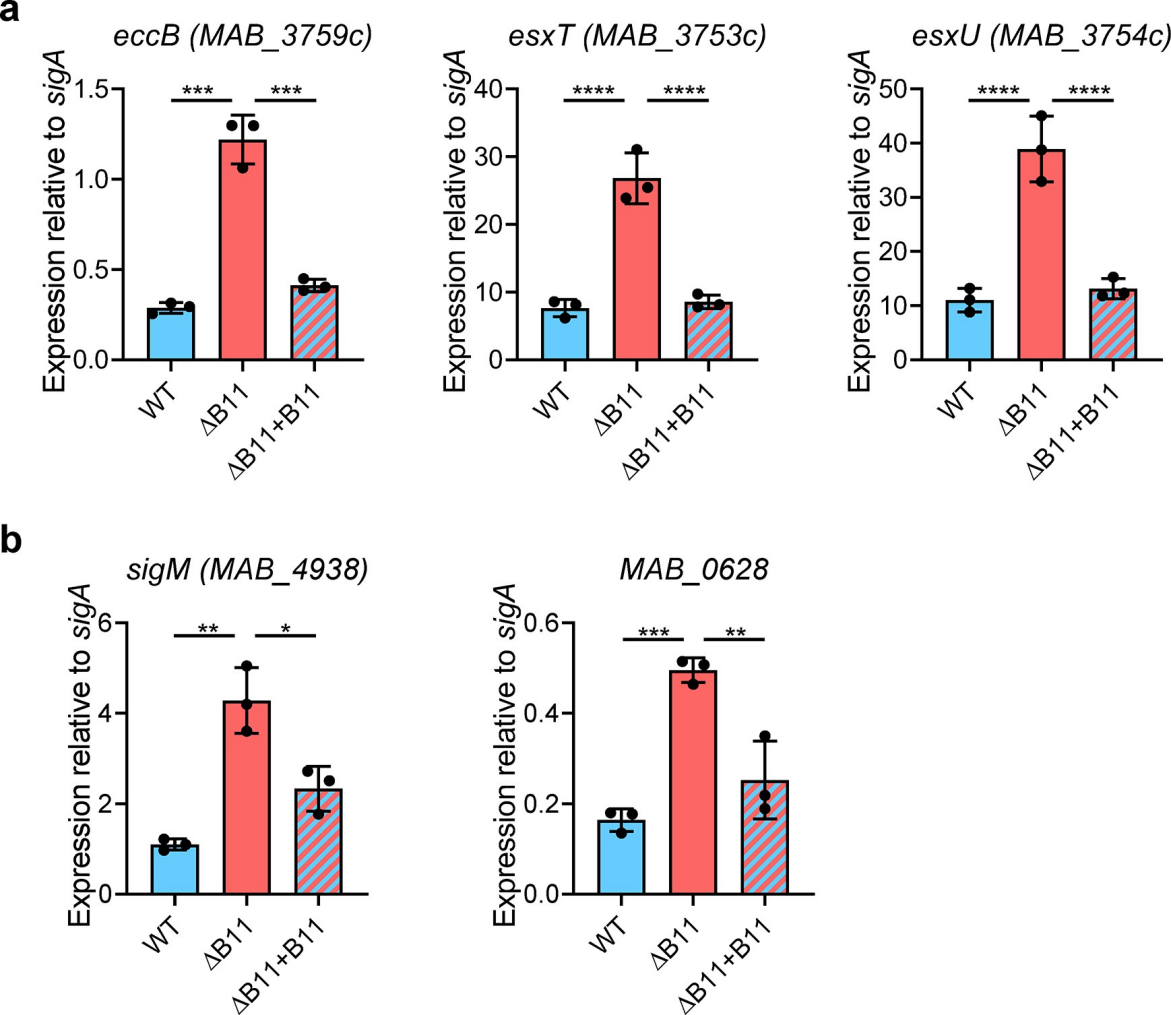
Disruption of *M*. *abscessus* B11 causes increased expression of several ESX-related genes with B11-complementary sequences. **A.** Disruption or deletion of B11 causes increased expression of genes encoding the EsxU, EsxT, and the ESX-4 structural protein EccB. **B.** Deletion of B11 causes increased expression of *sigM* and *MAB_0628*, which is predicted to be transcribed upstream of *espI* on a polycistronic transcript (see S4 Fig). Bars display means and SD. Black dots show the values for each sample. * *P* < 0.05; ** *P* < 0.01; *** *P* < 0.001; **** *P* < 0.0001, ANOVA with Tukey post-test. qPCR comparisons where *P* > 0.05 are not shown. qPCR data represent triplicate log-phase cultures. Data are representative of at least two independent experiments.

The genes differentially expressed by RNAseq included all eight genes reported to encode components of the ESX-4 secretion system and some possible ESX regulators [24]. Most of these either had B11-complementary sequences in their RBSs, or were in operons downstream of genes that had B11-complemantary sequence in their RBSs (S4 Fig). In one case, the gene was leaderless with a B11-complementary sequence early in the coding sequence (S4 Fig). To validate some of these expression changes, we performed qPCR on RNA from independently grown cultures. In most cases, deletion of B11 led to increased expression that was complemented when B11 was ectopically expressed (Fig 4A and 4B).

### B11 represses expression of a critical component of the ESX-4 secretion system via direct binding to its RBS

**Fig 5:**
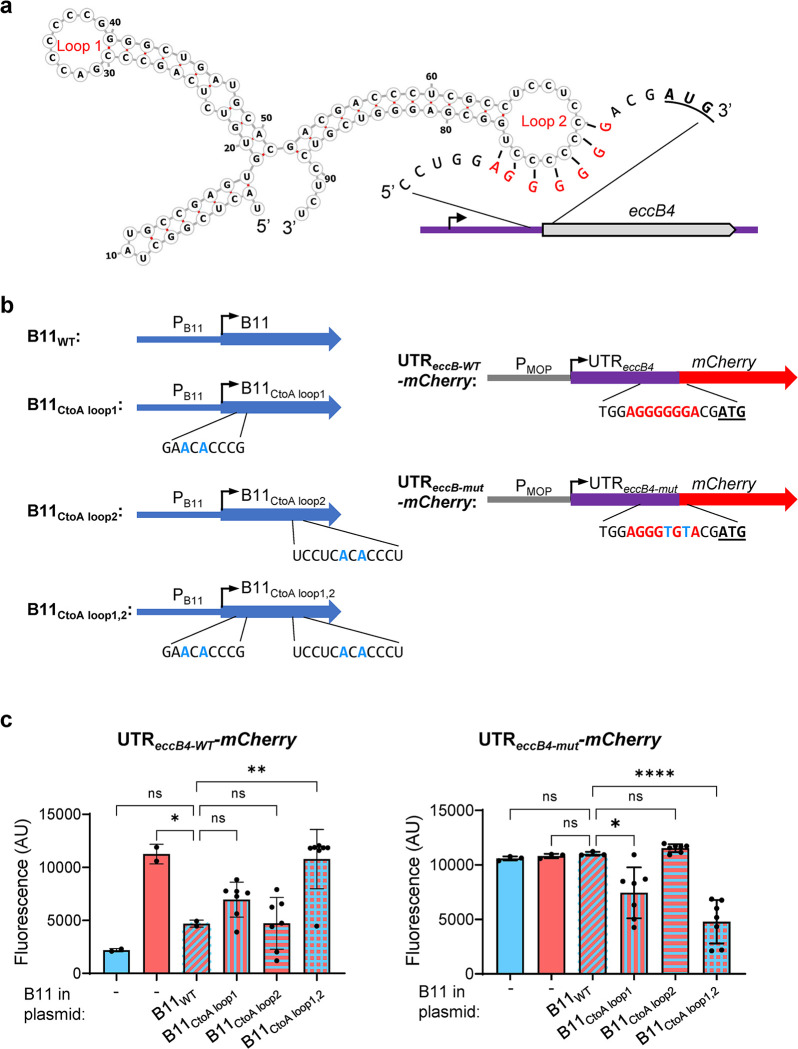
B11 represses expression of genes harboring the 5’ UTR of *M*. *abscessus eccB4* via direct binding. **A.** Schematic of the predicted secondary structure of B11 demonstrating the possibility of base-pairing to the ribosome binding site of the *eccB4* 5’ UTR. The *eccB4* start codon is underlined and bolded. Note that the 6-G sequence in the UTR could also base-pair with loop 1 of B11. **B.** Reporter constructs were built containing different versions of B11 expressed from its native promoter (left) and different versions of the *eccB4* 5’ UTR linked to the *mCherry* gene and expressed from the B11-insensitive MOP promoter. Mutated nts are indicated with bolded blue. The *eccB4* 5’ UTR sequence complementary to B11 is indicated in bolded red. Start codons are bolded and underlined. **C.** Reporter constructs containing the WT *eccB4* 5’ UTR (left) or the mutated version (right) along with the indicated version of B11 were transformed into WT or ΔB11 *M*. *abscessus* as indicated. mCherry fluorescence was measured by flow cytometry of biological replicate cultures. In each chart, the ΔB11 strain complemented with WT B11 was compared to the other strains by ANOVA followed by Dunnett’s multiple comparisons test. Bar charts display means and SD. Black dots represent individual samples. * *P* < 0.05; ** *P* < 0.01; **** *P* < 0.0001. Data are representative of two independent experiments.

One of the most strongly upregulated genes in the ΔB11 strain was *eccB4* (*MAB_3759c*), which was upregulated at both the mRNA and protein levels. EccB4 is one of the structural components of the ESX-4 secretion system and is required for *M*. *abscessus* growth in macrophages [24]. The *eccB4* 5’ UTR has a 7-nt sequence that is complementary to loop 2 of B11, as well as to 6 nt of loop 1 (Fig 5A). To test the hypothesis that B11 negatively regulates *eccB4* by binding to its 5’ UTR, we constructed a set of reporters in which mCherry was expressed from the synthetic MOP (a synthetic, constitutive, mycobacteria-optimized promoter, S6 Table) promoter either with the MOP-associated synthetic 5’ UTR (S5A Fig) or with the *eccB4* 5’ UTR (Figs 5B and S5C). mCherry expression from the construct with the MOP 5’ UTR was similar for the WT and ΔB11 strains (S5B Fig). In contrast, mCherry expression from the construct with the *eccB4* 5’ UTR was substantially higher in the ΔB11 strain compared to the WT and complemented strains (Fig 5C). The *eccB4* 5’ UTR contains a tract of six guanosines that could theoretically bind to either of B11’s two C-rich loops. To determine the roles of the B11 loops in the B11-mediated repression, we mutated each of the two loops individually and in combination (Fig 5B). B11 was still able to repress mCherry expression when either loop was mutated individually, but did not repress mCherry when both loops were mutated (Fig 5C). This suggests that either B11 loop is capable of repressing target gene expression.

To test if the B11-complementary sequence in *eccB4*’s UTR is needed for the B11-mediated repression, we then mutated the UTR to disrupt potential base-pairing with B11 (Fig 5B). Constructs containing the mutated *eccB4* UTR were insensitive to the presence or absence of WT B11 (Fig 5C). However, B11 variants with compensatory mutations in loop 1 or both loops were capable of repressing the constructs with the mutated UTR (Fig 5C). Taken together, these data indicate that the 5’ UTR of *eccB4* is sufficient to make a transcript B11-repressible via direct base-pairing with complementary sequences in the B11 loops.

### Loss or reduction of B11 function occurs in some clinical *M*. *abscessus* isolates

**Fig 6:**
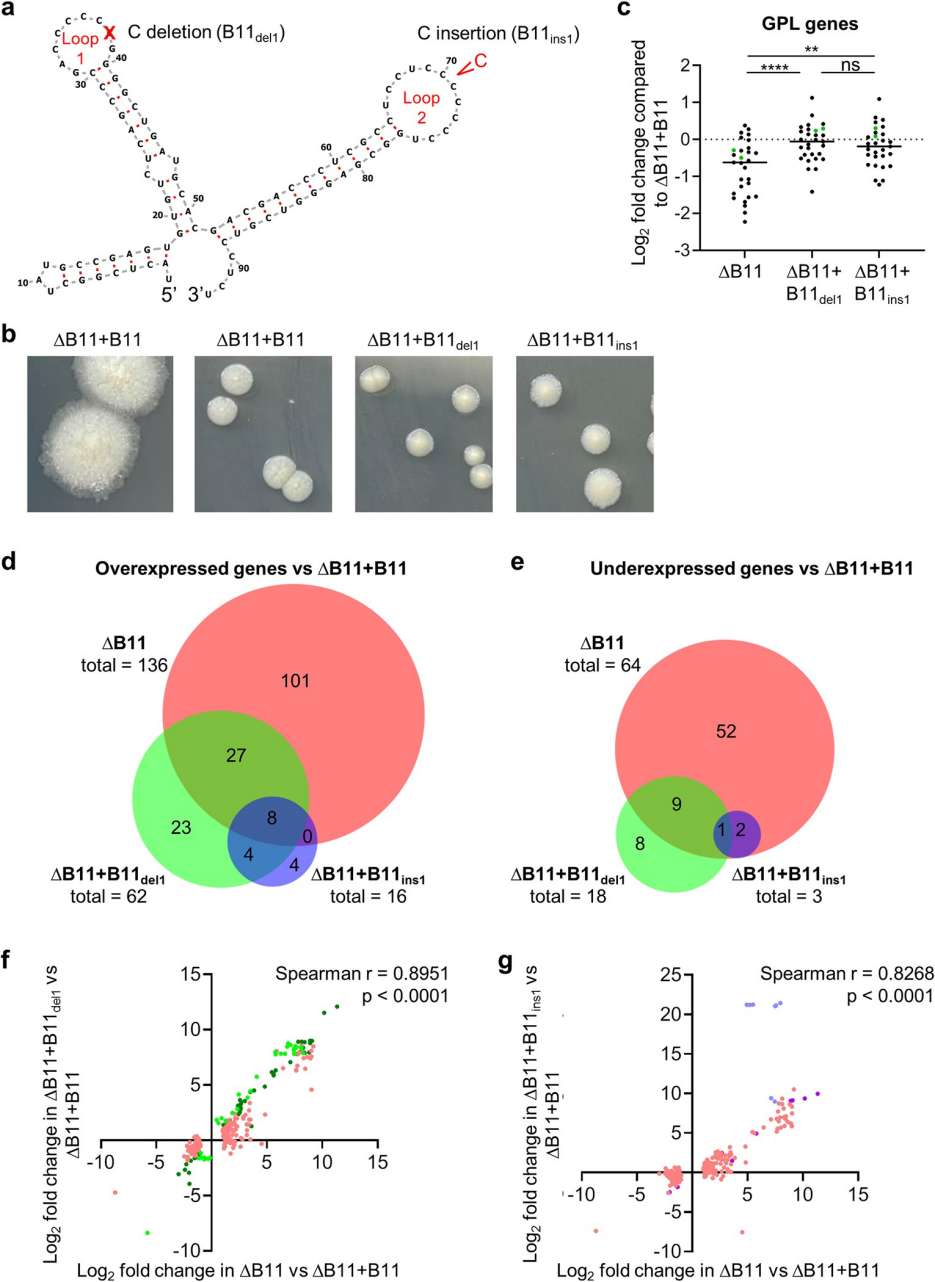
B11 mutations found in clinical *M*. *abscessus* strains cause partial loss of function. **A.** Deletion of one C from loop 1 was found in one clinical isolate, while insertion of one C into loop 2 was found in 3 clinical isolates. **B.** Smooth morphology was largely restored upon complementation of the ΔB11 strain with B11 harboring each of the two clinical loop mutations. **C.** RNAseq revealed that genes involved in GPL synthesis and transport had reduced expression in the ΔB11 strain and this was largely complemented by each of the clinical loop mutations. Each dot represents a gene associated with GPL metabolism (62). *mps1* and *mps2* are indicated with green dots. ** *P* < 0.01, **** *P* < 0.0001, one way RM ANOVA. **D-E.** Intersections of genes differentially expressed (log_2_ fold change > 1 or < -1, *P* < 0.05) in ΔB11/ΔB11+B11, ΔB11+B11_del1_/ΔB11+B11, and ΔB11+B11_ins1_/ΔB11+B11. Venn diagrams were created with BioVenn (101). *P* < 0.0001 for the overlaps between between all pairs of comparisons, hypergeometric test. **F.** There is a correlation between log_2_ fold change in genes differentially expressed in ΔB11/ΔB11+B11 and/or in ΔB11+B11_del1_/ΔB11+B11. Dark green dots represent genes differentially expressed in both comparisons. Pink dots represent genes that met the criteria for differential expression in ΔB11/ΔB11+B11 but not in ΔB11+B11_del1_/ΔB11+B11. Light green dots represent genes that met the criteria for differential expression in ΔB11+B11_del1_/ΔB11+B11 but not in ΔB11/ΔB11+B11. **G.** There is a correlation between log_2_ fold change in genes differentially expressed in ΔB11/ΔB11+B11 and/or in ΔB11+B11_ins1_/ΔB11+B11. Magenta green dots represent genes differentially expressed in both comparisons. Pink dots represent genes that met the criteria for differential expression in ΔB11/ΔB11+B11 but not in ΔB11+B11_ins1_/ΔB11+B11. Periwinkle dots represent genes that met the criteria for differential expression in ΔB11+B11_ins1_/ΔB11+B11 but not in ΔB11/ΔB11+B11.

Given the association between rough morphotypes and *M*. *abscessus* disease progression, we wondered if downregulation of B11 expression or activity occurred in clinical isolates as part of an *in-vivo* evolutionary process favoring progression to more virulent phenotypes. We examined a recently published cohort of 70 Irish *M*. *abscessus* clinical isolates that underwent whole genome sequencing [61]. Two of the seventy isolates had indel mutations in the B11 loops: one deletion of a C in the first loop (Shortening it from 6 Cs to 5 Cs; B11_del1_), and one insertion of a C in the second loop (from 7 Cs to 8; B11_ins1_; Fig 6A). We also examined a set of 52 clinical isolates from Denmark and found that two had the same B11_ins1_ mutation. To test the hypothesis that these mutations affect B11 stability or function, we complemented the ΔB11 strain with variants of B11 harboring the deletion in loop 1 or the insertion in loop 2. Both variants had similar abundance to WT B11 (S6 Fig), suggesting the mutations do not affect B11 expression or stability.

Complementation of the ΔB11 strain with B11_del1_ or B11_ins1_ largely restored smooth morphology, with subtle differences compared to WT B11 (Fig 6B). Approximately half of the GPL biosynthesis and transport genes (MAB_4117c-MAB_4097c, MAB_0934-MAB_0939, MAB_4437, MAB_4454c, MAB_4459c, and MAB_4633, as reported in [62]), were underexpressed in the ΔB11 strain, and their expression was largely restored by both B11 loop mutants (Fig 6C, S2 Table). This suggests that the B11 clinical variants B11_del1_ and B11_ins1_ do not affect GPL gene expression and colony morphology relative to WT B11.

To further assess the functional impact of the two clinical loop mutations, we performed RNAseq on the ΔB11 strain complemented with each of the two mutated version of B11, B11_del1_ and B11_ins1_ (S1 Table). We compared the ΔB11 strain complemented with B11_del1_ or B11_ins1_ (ΔB11+B11_del1_ and ΔB11+B11_ins1_) to the ΔB11 strain complemented with WT B11 (ΔB11+B11). This allowed us to cleanly assess the impact of the two B11 mutations without being confounded by effects of the gene deletion and complementation methods. We found that ΔB11+B11_del1_ had 80 differentially expressed genes compared to ΔB11+B11, of which 45 were also differentially expressed in ΔB11 compared to ΔB11+B11 (Fig 6D and 6E and S3 Table). This represents a significant overlap in differentially expressed genes in ΔB11 and ΔB11+B11_del1_ (*p* < 0.0001, hypergeometric test). The ΔB11+B11_ins1_ strain had 19 differentially expressed genes compared to ΔB11+B11, of which 11 were also differentially expressed in ΔB11 compared to ΔB11+B11 and 13 were also differentially expressed in ΔB11+B11_del1_ compared to ΔB11+B11 (Fig 6D and 6E and S3 Table). The overlap between ΔB11+B11_ins1_ and ΔB11 was statistically significant, as was the overlap between ΔB11+B11_ins1_ and ΔB11+B11_del1_ (*p* < 0.0001 for both comparisons, hypergeometric test).

Similarities in the gene expression changes in each of the mutant versions of B11 compared to complete deletion of B11 were further revealed by scatterplots comparing the log_2_ fold changes of genes differentially expressed in these strains (Fig 6F and 6G). Many genes that were differentially expressed in the ΔB11 strain also had similar expression trends in ΔB11+B11_del1_, despite many of them not meeting the thresholds for statistical significance (Fig 6F). Similarly, many genes that were differentially expressed in the ΔB11 strain also had similar expression trends in ΔB11+B11_ins1_, while not meeting the criteria for statistical significance (Fig 6G). Together, the data suggest that these two B11 mutations found in clinical isolates of *M*. *abscessus* may be hypomorphic, causing partial loss of B11 function. The ESX-4-related genes shown in Fig 4 did not appear to be overexpressed in the strains expressing B11_ins1_ or B11_del1_, and most of the differentially expressed genes in these mutants did not have B11-complementary motifs. This suggests that many of the expression changes may be indirect, or that B11 may have additional modes of action besides direct base-pairing to RBS regions in targets. The genes that were differentially expressed in ΔB11, ΔB11+B11_del1_, and ΔB11+B11_ins1_ included four overexpressed genes (*MAB_0894c*, *MAB_0895c*, *MAB_0902*, and *MAB_0904*) predicted to participate in catabolism of odd-chain and branched-chain fatty acids, which are common carbon sources for mycobacteria in host environments. Another pattern that emerged was underexpression of genes encoding three protein chaperones (GroS, GroL, and DnaK) in both ΔB11 and ΔB11+B11_del1_.

Examination of three additional sets of publicly available clinical *M*. *abscessus* genome sequences totaling 750 patients revealed 17 additional isolates with B11 mutations (S4 and S5 Tables), indicating a similar frequency of B11 mutations as observed in the Danish and Irish datasets (~2.3–2.5%) [63–70]. While some additional loop mutants were observed, the B11_del1_ and B11_ins1_ mutations described above were two of the three most prevalent types.

**Fig 7:**
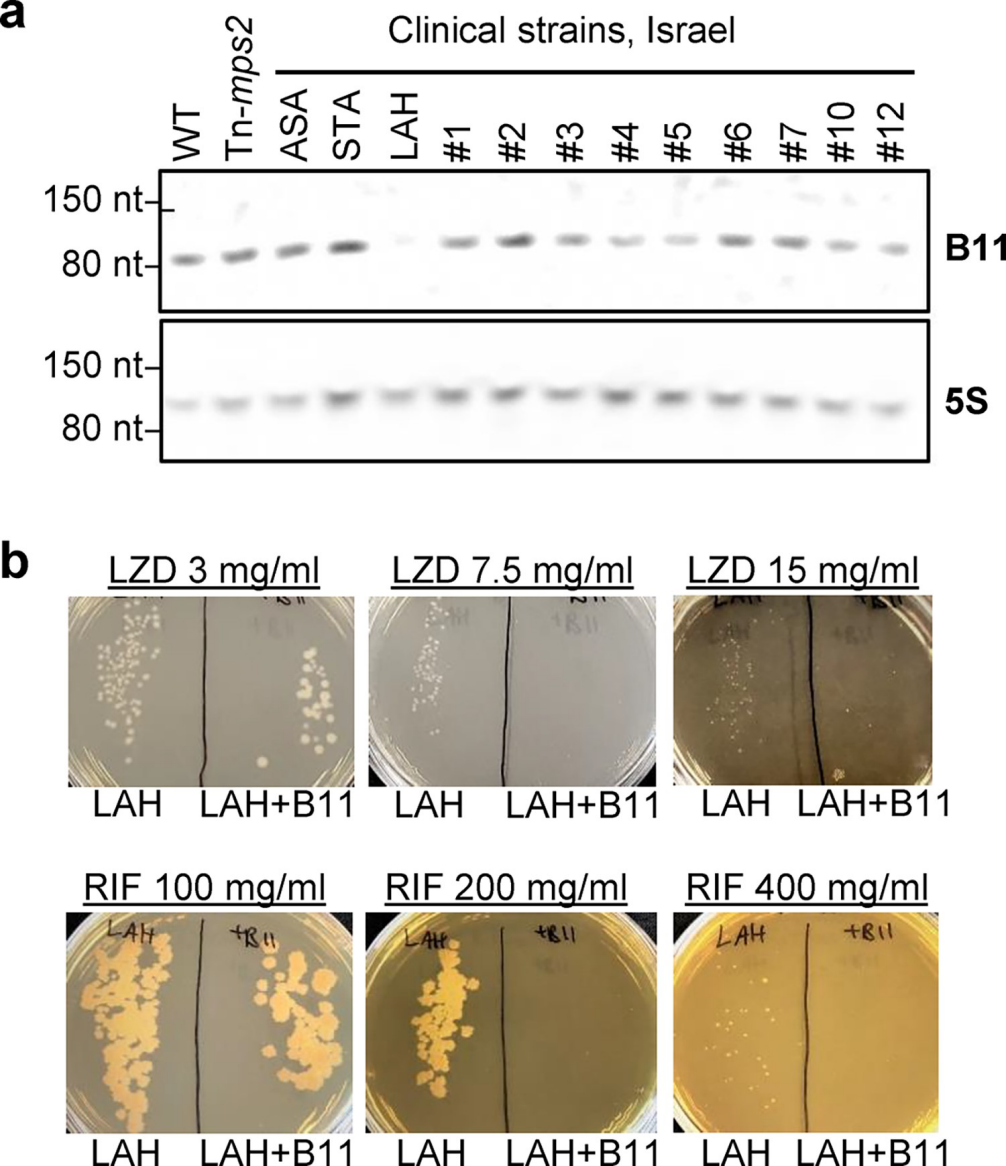
A clinical *M*. *abscessus* strain does not express B11 and had increased antibiotic sensitivity upon ectopic B11 expression. **A.** Northern blot for B11 in log phase cultures of WT *M*. *abscessus*, a transposon-mutant of *mps2* (MAB_4098c), and twelve clinical isolates from an Israeli cohort. **B.** The clinical isolate LAH was electroporated with a single copy plasmid encoding B11 under the MOP promoter, and sensitivity to linezolid and rifampin was examined. Data are representative of two independent experiments.

Finally, we examined B11 expression in a set of clinical isolates from Israel, and found that one isolate, termed LAH, had a dramatic reduction in B11 abundance compared to ATCC_19977 and the other clinical strains (Fig 7A). Sequencing of B11 and the surrounding region did not reveal any mutations that could explain why B11 had reduced expression in LAH, suggesting *trans*- regulatory changes. To assess the functional impact of the absence of B11 in this strain, we transformed it with a plasmid to ectopically express B11 from a constitutive artificial promoter (MOP) and measured resistance to linezolid and rifampicin. Consistent with our finding that deletion of B11 from ATCC_19977 increased resistance to these antibiotics, ectopic expression of B11 caused increased sensitivity in LAH (Fig 7B). Taken together, our findings suggest that mutations and regulatory changes that either abrogate the expression, or affect the biologic potency of the B11 transcript, are found in clinical isolates throughout the globe.

## Discussion

In most bacteria, virulence mechanisms (such as ESX-4 in *M*. *abscessus*) and virulence-related phenotypes (such as the lack of GPL in *M*. *abscessus*, leading to a rough colony-morphology phenotype) are tightly regulated at multiple levels. Small non-coding RNAs have been shown to play an important role in such regulation in several bacteria, where their role is usually of "fine tuning", rather than overt "all or none" regulation. In mycobacteria, only limited data exist on physiologic roles of sRNA, and even fewer are directly linked to virulence. The mechanisms of genetic regulation by sRNAs are also understood to a lesser extent in mycobacteria than in other bacteria, partially due to a lack of identified RNA-chaperone proteins (such as Hfq and ProQ). In this work, we identified that in *M*. *abscessus* the sRNA B11 affects expression of over 200 genes. B11 appears to negatively regulate some genes directly, while positively regulating others, possibly through indirect mechanisms. Further study is needed to determine which genes are regulated directly at the level of transcript stabilization or destabilization and which are regulated indirectly at the level of transcription. Interestingly, B11 seems to negatively regulate one virulence system (the ESX-4) and positively regulate GPL production (which may be considered an "anti-virulence" system)–collectively making it a suppressor of pathogenesis. Deletion mutants were hyper-virulent in several experimental models (cell-culture, infection of larvae, and a murine model of lung infection).

If B11 is indeed a negative regulator of virulence, a question arises whether B11 downregulation plays a role in the clinical course of human infections. Most patients with CF who eventually develop severe *M*. *abscessus* lung disease are first colonized by smooth variants. Over time, mutations favoring virulence arise, are naturally selected by the host environment, and accumulate in the *M*. *abscessus* population. Chronic inflammatory disease ensues–to a great extent due to a destructive immune response of the host. This process entails a different set of genetic events in each clinical isolate of *M*. *abscessus*. By examining cohorts of over 100 clinical isolates from Ireland, Denmark, and Israel we found one isolate with substantial downregulation of B11 expression, and several isolates with indel mutations in the C-loops of B11 –mutations we show to reduce the biologic activity of the molecule. Taken together, our findings are consistent with the idea that downregulation of B11 (either through expression or mutations) may be one of the many genetic mechanisms and events by which *M*. *abscessus* evolves *in-vivo* towards a more virulent phenotype. The small but consistent increases in the bacteria’s MIC to linezolid and rifampicin after B11 deletion also stress the role this molecule may play in exacerbating the clinical picture in affected patients.

It is notable that while we defined a role for B11 as a regulator of translation, we observed changes in mRNA levels for over 200 genes. Some genes had altered expression levels as a consequence of the process of generating the ΔB11 strain; these included two genes downstream of the B11 locus, which are likely overexpressed due to transcriptional readthrough from the zeo resistance cassette used to replace B11, and the gene upstream of the B11 locus, which also appeared to be overexpressed due to the presence of the zeo resistance cassette. The B11 deletion mutant also lost a naturally occurring mercury resistance plasmid present in ATTC_19977 [71]. However, 217 genes had differential mRNA levels in the ΔB11 strain that were complemented by ectopic expression of B11 from an integrating plasmid, indicating that their differential expression was attributable to the absence of the B11 sRNA. There are at least three mechanisms by which B11 is likely to affect mRNA abundance as a consequence of repressing translation.

First, impediments to ribosome binding or translation often lead to faster mRNA degradation in bacteria [53,55,56,58] and there is a positive correlation between translation efficiency and mRNA half-life [72]. B11 targets may therefore be degraded more slowly when B11 is deleted, leading to higher steady-state mRNA abundance. Second, translational blocks often lead to premature transcriptional termination in bacteria due to Rho-mediated termination ([57] and reviewed in [73]). Bacterial translation begins shortly after the portion of an mRNA containing the ribosome binding site is synthesized, and the processes of transcription and translation are thus coupled in space and time [54]. The closeness of this coupling varies among the bacteria, and mycobacterial gene structures are indicative of close coupling [74]. Rho terminates transcription by binding to regions of exposed mRNA and translocating to RNA polymerase where it triggers dissociation of the polymerase from the DNA and RNA (reviewed in [75]). The absence of ribosomes adjacent to the RNA polymerase therefore sensitizes transcripts to premature Rho-mediated transcriptional termination (reviewed in [73]). Rho-dependent termination is prevalent in the mycobacteria [76–78], and translational block by B11 binding is therefore expected to lead to premature transcriptional termination and thereby lower steady-state mRNA abundance of many B11 targets. Transcription of B11 target genes is therefore expected to be higher in the ΔB11 strain. Third, the altered expression of B11 targets is expected to have downstream effects of expression of other genes. For example, two predicted transcriptional regulators, MAB_0208 and MAB_2602c, are predicted to be direct targets of B11 due to having defined 5’ UTRs containing B11-complementary sequences and having increased mRNA abundance in the ΔB11 strain that was complemented in the ΔB11+B11 strain. MAB_0208 also had increased protein abundance in the ΔB11 strain, while the proteomics data did not allow a high-confidence assessment of MAB_2602c. The increased expression of these two transcriptional regulators in the ΔB11 strain likely affects transcription of their regulons. Other direct targets of B11 that are not transcriptional regulators may also affect expression of other genes by altering cellular physiology.

As we mentioned, the mechanisms by which sRNAs act in mycobacteria, in the apparent absence of an sRNA chaperone, are poorly understood. In [42], examination of the interaction between the B11 homolog of Mtb with genes of *M*. *smegmatis* led to the suggestion that direct pairing of bases could occur without need of a chaperone. This work also suggested that B11 may be essential, as a deletion mutant could not be created in *M*. *smegmatis*. Our results are not in accordance with the latter hypothesis, as B11 deletion mutants were relatively easily constructed both in *M*. *abscessus* and in *M*. *smegmatis*. However, our results do agree with the hypothesized direct interaction of either one of the long C-loops with G-rich areas in the UTRs of genes regulated by B11; mutation of the loops or the UTR individually prevented B11-mediated repression, but simultaneous mutation of the loops and UTR to restore base-pairing also restored repression. Both RNAseq and whole cell proteomic analyses showed genes with long G-stretches in their UTRs are much more likely to be affected by B11 than genes without these G-stretches in the UTRs.

On the surface it may seem paradoxical that we found a modest enrichment for B11-complementary sequences in the RBS regions among genes with lower mRNA abundance in the ΔB11 strain (Fig 3G). However, there is evidence from various bacteria that in some cases, secondary structure or other physical blocks near the 5’ ends of transcripts, including sRNAs, can block degradation and thus lengthen transcript half-life [55,79–84]. Further study will be needed to understand the mechanism by which some genes with B11-complementary motifs were underexpressed upon B11 deletion.

Previously, researchers performing a transposon-mutant screen in *M*. *kansasii* found a transposon insertion at the equivalent TA position to our Tn-B11 mutant [43]. However, loss of B11 caused small colony size and a smoother morphology in *M*. *kansasii*, in sharp contrast to our results. This suggests that B11 plays different roles in *M*. *kansasii* and *M*. *abscessus*. It is possible that while the structure and mechanism of translational repression by B11 are conserved among mycobacteria, the pathways being regulated have diverged among species. Budell *et al* [43] also tested pathogenesis in the *G*. *mellonella* model but found no difference, whereas we found the B11 mutant was hypervirulent. This could reflect further differences in the roles of B11 in the two species. Alternatively, they could be due to experimental design; Budell *et al* [43] were looking for attenuation and therefore designed the experiment such that the WT control produced maximal virulence, making hypervirulence in a mutant hard to detect. In our study we used a smaller inoculum, allowing the hypervirulent phenotype to be uncovered.

The interplay of B11 with the ESX-4 system is intriguing. ESX-4 secretion was shown some time ago to play an important role in *M*. *abscessus* pathogenesis [24]. Here we showed that B11 deletion causes upregulation of the expression of ESX-4 components, including the structural gene *eccB4*, and the two secretion substrate genes *esxU* and *esxT*. This initially suggested that part of the explanation for the increased virulence could be increased ESX-4 activity. However, a recent paper found that specific deletion of the secretion substrates alone (*esxU* and *esxT*) caused a hypervirulent phenotype in mice, suggesting the role of ESX-4 in virulence is complex and still poorly understood [85]. The exact interplay, therefore, of B11, ESX-4 as a whole, specifically the ESX-4 secretion substrates and bacterial virulence should continue to be a focus of intense research.

## Materials and methods

### Bacteria and growth conditions

*M*. *abscessus* ATCC_19977 was obtained from the ATCC collection. Clinical isolates were obtained from the Israeli Ministry of Health Mycobacteria Central Laboratories, and from clinical microbiology laboratories in various hospitals. Bacteria were grown in 7H9 media supplemented by 0.05% glycerol, 10% ADC or OADC, and tween 80, as widely described. For solid agar plates, either LB supplemented with 0.05% glycerol and 0.05% dextrose was used, or 7H10 plates with glycerol and ADC or OADC. Antibiotic concentrations were: kanamycin 120–240 μg/ml and zeocin 33–50 μg/ml for *M*. *abscessus*; kanamycin 40 μg/ml and zeocin 33 μg/ml for *E*. *coli*.

### Creation of a Tn-mutant library and identification of the Tn-insertion site in selected clones

To construct the transposon, the zeocin and kanamycin resistance genes were cloned next to each other, with the 27 bp of the Himar-1 inverted repeat on either side. The transposase was PCR amplified from MycoMar7 phage (kind gift from Eric Rubin and Chris Sassetti), and cloned next to the transposon, but outside of the IR. This construct was used to create a temperature-sensitive, TM4-based mycobacteriophage, as previously widely described [86]. This phage was used to infect *M*. *abscessus* at 37°C, and bacteria were then plated on 7H10 plates supplemented with zeocin (50 μg/ml) and kanamycin (120 μg/ml). Aberrant-appearing colonies were identified by the naked eye, and analyzed individually. To identify the Tn-insertion site in clones of interest, genomic DNA was extracted, digested by SalI, self-ligated, and a PCR amplifying the genome-IR junction was performed Using primers M6 and M5 (see S6 Table). The resulting fragment was sent for Sanger sequencing, identifying the Tn-junction area. Strain names are listed in S7 Table.

### RNA extraction, northern blot, and evaluation of gene expression by qPCR

*M*. *abscessus* log phase cultures were used to inoculate 50 ml conical tubes containing 10 ml of 7H9 medium to an OD_600_ = 0.01. Cultures were grown at 37°C and 200 RPM until they reached an OD_600_ between 0.6–0.8 and were frozen with liquid nitrogen and stored at -80°C until RNA purification. RNA was extracted as in [87]. Briefly, frozen cultures were thawed on ice and centrifuged at 4,000 rpm for 5 min at 4°C. The pellets were resuspended in 1 ml Trizol (Life Technologies) and placed in tubes containing Lysing Matrix B (MP Bio). Cells were lysed by bead-beating twice for 40 s at 9 m/sec in a FastPrep 5G instrument (MP Bio). RNA was purified using Direct Zol RNA miniprep kit (Zymo) according to manufacturer’s instructions. RNA concentrations were determined using a Nanodrop instrument. RNA samples were stored at -80°C until use.

The relative abundance of B11 transcript among strains was evaluated by northern blot as follows: 5 μg of each RNA sample (or 1 μg for 5S, used as a load control) were mixed with TBE-Urea sample buffer (Novex) and run in a 10% TBE-Urea Polyacrylamide gel (Biorad) at 180 V for 1 hour. RNA was transferred to a positively charged nylon membrane (Amhersham) during 80 min at 10 V and 400 mA and then RNA was cross-linked by exposure to UV light (302 nm) for 7 min. Prior to probe hybridization, the membrane was incubated with 10 ml of ULTRAhyb buffer (Ambion) at 50 °C for 30 min. Then, the membrane was incubated with 10 ml of ULTRAhyb buffer containing ~ 200 ng of B11 RNA-probe or 5S RNA-probe (see S6 Table) at 50°C overnight. The membrane was washed with 30 ml of low stringency wash solution (2X SSC, 0.1% SDS) for 10 min at room temperature, then washed with 30 ml of high stringency wash solution (0.1 SSC, 0.1% SDS) at 50°C for 15 min and finally washed with 20 ml washing buffer (Roche) at room temperature for 5 min. The membrane was incubated with 20 ml of blocking solution (Roche) for 30 min and then incubated with 20 ml of antibody solution (Roche) containing 1 μL of Anti-digoxigenin-AP conjugate (Roche) at room temperature for 30 min. The membrane was washed twice with 20 ml of washing buffer and then incubated with 20 ml of detection buffer for 3 min. Detection was done by incubation with 1 ml of detection buffer containing 100 μL CDP-Star (Roche) and exposure in a Gel Doc.

Expression of *MAB_3759c*, *MAB_3753c*, *MAB_3754c*, *MAB_0498c*, *MAB_0499c*, *MAB_0628*, and *MAB_4938* relative to *sigA* was determined by qPCR using the primers listed in S6 Table. cDNAs were prepared as described [87]. For each qPCR reaction, iTaq SYBR green (Bio-Rad) was mixed with 200 pg cDNA and 0.25 μM each primer in 10 μl reaction mixtures. The qPCR parameters were: 40 cycles of 15 s at 95°C and 1 min at 61°C.

### RNAseq

rRNA was depleted and paired-end Illumina sequencing libraries were constructed as described [88,89]. Libraries were sequenced at the UMass Medical School Deep Sequencing Core Facility on a HiSeq 2000. Raw fastq files were demultiplexed using Cutadapt [90]. by providing corresponding sample barcodes. Reads were mapped to the NC_010397 reference genome using Burrows-Wheeler Aligner mem [91]. The FeatureCounts tool was used to assign mapped reads to genomic features for producing count matrices [92]. Principal Component Analysis (PCA) was applied for quality assessment of RNA expression libraries (S7 Fig). Deseq2 [93] was used to assess changes in gene expression. The Deseq2 package internally corrects for library size. The input count matrix for differential expression analysis were un-normalized counts, which allow assessing the measurement precision correctly. Raw and processed data are available in GEO, accession number GSE214640.

### Targeted B11 deletion in *M*. *abscessus*

*M*. *abscessus* ATCC19977 was first electroporated with the plasmid pJV53 (kanamycin), with recombineering enzymes under an acetamide-regulated promoter [94], to enhance targeted deletion events when using a specialized TM4-based mycobacteriophage [95]. The 5’ and 3’ flanking regions (600 bp long) of B11 were cloned on either side of the zeocin^R^ gene, to create pDB386. This plasmid was used to create the specialized transducing phage phDB35 (86). *M*. *abscessus*^pJV53^ was then grown in media with 0.2% succinate and no dextrose, induced by acetamide for 4 hours, and infected by phDB35 for 3 hours at 37°C. Bacteria were then washed, re-suspended in 7H9 media, and plated on 7H10 plates with 50 μg/ml zeocin. One of multiple zeocin-resistant colonies was further examined by PCR reactions with the primers yielding a 1500 bp product in WT and a 1900 bp product in a mutant where a correct replacement took place (S1C Fig) Sanger sequencing of the product was also performed. After confirmation of the deletion, the mutant was grown and plated for single colonies. Colonies were then patched with and without kanamycin, to verify the loss of pJV53. A kanamycin-sensitive colony was isolated, and the final strain, *M*. *abscessus* ΔB11, was named mDB228.

### Complementation of the B11 deletion in *M*. *abscessus*

The region spanning from 250 bp upstream to 200 bp downstream of B11 was PCR-amplified and cloned into the L5 *attB*-integrating, kanamycin-selected plasmid pDB213, to create pDB392. This plasmid was used to complement the ΔB11 mutant (mDB228), to create a complemented mutant mDB252 (ΔB11^zeo^+B11^kan^). Specifically for the *G*. *mellonella* infection experiment described in Fig 2B, the complementation was done by a similar plasmid, but where the integrase gene was interrupted to increase integration stability in conditions not allowing continuous selection by kanamycin. PCR-mutagenesis was used to introduce a C deletion and a C insertion in the first C-rich loop and second C-rich loop of B11, respectively, in pDB213 (Fig 6A), and the resulting plasmids were transformed into mDB228, to create the complemented mutants mDB281 (ΔB11^zeo^+B11_del1_^kan^) and mDB282 (ΔB11^zeo^+B11_ins1_^kan^). PCR-mutagenesis was also used to introduce multiple base substitutions into the second C-loop of B11 (S1E Fig), to create mDB269 (ΔB11^zeo^+B11_mutated_^kan^).

### Construction of the reporter systems

For the reporter system described in Fig 5, *M*. *abscessus* ATCC_19977 and the ΔB11 mutant were electroporated with a set of reporters where mCherry was expressed under the MOP promoter either with the 55 nt that correspond to the MOP-associated synthetic 5’ UTR (P_MOP__UTR_MOP__mCherry, S5 Fig.) or with the 74 nt that correspond to the *eccB4* 5’ UTR (P_MOP__UTR_eccB4__mCherry) (Fig 5B and 5C). Then, to evaluate which loop of B11 was responsible for its mediated regulation, different versions of B11 were cloned into the P_MOP__UTR_eccB4__mCherry construct divergently from P_MOP_. PCR was used to amplify B11 and its native promoter (223 nt upstream B11) and the obtained fragment was cloned into the P_MOP__UTR_eccB4__mCherry reporter system to generate the B11_P_B11__B11_P_MOP__UTR_eccB4__mCherry construct. PCR-mutagenesis was later used to introduce multiple versions of base substitutions into the first, second or both loops, and/or into the *eccB4* 5’ UTR, as described in the text (Fig 5B–5D). All constructs were done on kanamycin-selected L5-integrating plasmids.

### Flow cytometry

Three to seven biological triplicates of each strain were used to evaluate mCherry expression by flow cytometry. Cultures were grown in 7H9 media with glycerol to an OD_600_ of 0.5, 2 ml of cultures were centrifuged, and the bacterial pellets were resuspended in 4% paraformaldehyde (Thermo Scientific). After 1 h of incubation at room temperature cultures were washed twice with PBST (PBS + 0.1% Tween 20, freshly filtered with a 0.2 μm filter to remove precipitates that otherwise appear as bacteria-like events on the flow cytometer) and resuspended in 2 mL of freshly filtered 7H9 media with glycerol. Cultures were filtered using 5 μm filter needles (BD Nokor filter needle) to remove clumps and a final dilution using freshly filtered 7H9 media with glycerol was done to achieve a final concentration of OD = 0.025. Samples were analyzed with a Cytoflex S (Beckman Counter) flow cytometer. The parameter settings were for Gain: FSC 500, Violet SSC 50 and for Thresholds: Violet SSC-H 100,000, FSC-H 10,000. Filtered 7H9 media was used to wash between samples and 5000 events were recorded within a gate selected to capture individual cells. Data were analyzed using the FlowJo V10 Software and the median of the detected fluorescence was compared between strains.

### Proteomic analysis of *M*. *abscessus* mutants

For proteomic analysis of bacteria, *M*. *abscessus* was grown in 7H9 media with glycerol but no albumin to an O.D_600_ of 0.3, 10 ml of cultures were centrifuged, and the bacterial pellet was stored at -80°C. Sample preparation and LC-MS/MS were performed at the Smolar Centre for Proteomics, Technion, Haifa, Israel as follows: The samples were mixed with 8 M Urea, 100 mM ABC, and 2.8 mM DTT (60°C for 30 min), modified with 8.8 mM iodoacetamide in 100 mM ammonium bicarbonate (in the dark, room temperature for 30 min) and digested in 2 M Urea, 25 mM ammonium bicarbonate with modified trypsin (Promega) at a 1:50 enzyme-to-substrate ratio, overnight at 37^o^C. An additional second digestion was done for 4 hours. The resulting peptides were desalted using C18 tips (Homemade stage tips) dried and re-suspended in 0.1% Formic acid**.** The peptides were resolved by reverse-phase chromatography on 0.075 X 180-mm fused silica capillaries (J&W) packed with Reprosil reversed phase material (Dr Maisch GmbH, Germany). The peptides were eluted with a linear 60 minute gradient of 5 to 28%, 15 minutes gradient of 28 to 95%, and 15 minutes at 95% acetonitrile with 0.1% formic acid in water at flow rates of 0.15 μl/min. Mass spectrometry was performed by Q Exactive plus mass spectrometer (Thermo) in a positive mode using repetitively full MS scan followed by collision induces dissociation (HCD) of the 10 most dominant ions selected from the first MS scan.

The mass spectrometry data were analyzed using the Protein Discoverer 1.4 (Thermo) using Sequest search engine, searching against *M*. *abscessus* of the Uniprot database. Peptide- and protein-level false discovery rates (FDRs) were filtered to 1% using the target-decoy strategy.

### Measurement of GPL content

*M*. *abscessus* strains grown on 7H10-ADC agar were extracted twice with water-saturated 1-butanol for 2 hours at room temperature. The 1-butanol extracts were pooled and dried under nitrogen, and GPLs were recovered by partitioning between 1-butanol and water (1:1, v/v). The top 1-butanol fraction containing GPLs was dried, resuspended in CHCl3/CH3OH (2:1, v/v) and resolved by TLC or LC-MS in positive ion mode following the method described by Sartain et al. [13] on a high-resolution Agilent 6546A TOF mass spectrometer interfaced to an LC.

### Protein secretion assay

*M*. *abscessus* ATCC_19977, ∆B11 mutant, and ΔB11+B11 were grown to an OD_600_ of 0.8 in 7H9 media with glycerol, washed twice with PBS, resuspended in Sauton media (KH_2_PO_4_ 0.5 g, MgSO_4_.7H_2_O 0.5 g, citric acid 2.0 g, ferric ammonium citrate 0.05 g, glycerol 60 ml, asparagine 4.0 g per L) supplemented with 0.05% Tween 80 and grown for 24 hours to an OD_600_ of ~0.6–0.8. Cultures were pelleted, and whole cell lysates were made by resuspending the pellets in 500 μl protein extraction buffer (50 mM Tris pH 7.5, 5 mM EDTA, and 1X protease inhibitor cocktail with 0.5 M EDTA), transferring to lysing matrix B tubes (MP Bio), and disrupting by bead-beating in a Fastprep 5G instrument (MP Bio) for 4 cycles of 40 seconds at 9 m/sec and 2 min incubations on ice between cycles. After cell disruption, 170 μl of SDS-PAGE loading buffer (200 mM Tris pH 6.8, 400 mM DTT, 8% SDS, 0.4% bromophenol blue, 40% glycerol) was added to whole cell lysates.

In parallel, culture supernatants were combined with 100 μl protease inhibitor cocktail (Thermo Scientific) with 0.5 M EDTA, filtered through 0.2 μm pore size filters (Genesee Scientific), and incubated overnight with 5 mL of concentrated trichloroacetic acid (Chemicals BDH) at 4°C. Then proteins were pelleted by 20 minutes of centrifugation at 14,500 RPM at 4°C, washed once with 100% acetone (Chemicals BDH), centrifuged, and finally resuspended in 100 μl protein extraction buffer plus 100 μl SDS-PAGE loading buffer. Both whole cell lysate and culture supernatant samples were heated at 95°C for 5 minutes before running on 4–20% PAGE gels in tris-glycine-SDS buffer and staining with Coomassie Blue.

Protein secretion assays were also performed using *M*. *abscessus* ATCC19977, ∆B11 mutant, and tn-*mps2* transformed with a plasmid expressing mCherry (P_MOP__UTR_MOP__mCherry). Whole cell lysate and supernatant of two biological replicates were probed by western blotting using a 1:1000 dilution anti-mCherry antibody (Proteintech) as a primary antibody and a 1:30000 dilution goat anti-rabbit antibody as a secondary antibody. Detection was performed by incubation with Radiance ECL (Azure biosystems) according to manufacturer’s instructions and exposure in a Gel Doc.

### Infection of RAW cells and TNF secretion measurement

RAW 264.7 macrophages were cultured in DMEM (ATCC) supplemented with 10% fetal bovine serum. Prior to infection, RAW 264.7 cells were seeded in 6-well plates (2 x 10^5^ per well). For experiments on exposure to heat-killed bacteria, heat-killed (30 minutes in 65°C) *M*. *abscessus* ATCC_19977 and ∆B11 mutant were prepared. RAW 264.7 were exposed to the bacteria at a multiplicity of infection (MOI) of 20. After incubation for 4 hours, cells were washed with phosphate-buffered saline (PBS) three times and fresh culture medium was added. Following 24 hours of incubation, culture medium was collected and analyzed for murine TNF by ELISA kit (Invitrogen, 88–7324) according to manufacturer’s instructions. For live-bacteria infection experiments, RAW cells were prepared and seeded the same way. Live *M*. *abscessus* ATCC_19977 and ∆B11 mutant were prepared and used to infect the RAW cells at an MOI of 5. After an incubation of 6 hours, the culture medium was collected for TNF measurement by a similar ELISA kit.

### Murine infections

Animal studies were conducted according to protocols and adhering strictly to the Italian Ministry of Health guidelines for the use and care of experimental animals (IACUC N°816) and approved by the San Raffaele Scientific Institute Institutional Animal Care and Use Committee (IACUC). Immunocompetent C57BL/6NCrl male mice (8 to 10 weeks of age) from Charles River were used for all experiments. Mice were maintained in specific pathogen-free conditions at San Raffaele Scientific Institute, Milan, Italy. *M*. *abscessus* ATCC_19977 and the ∆B11 mutant were streaked on 7H10 plates and colonies used to inoculate 20 ml of Middlebrook 7H9 broth. After two days of growth, the bacteria were embedded in agar beads as previously described [50,51,96], mice were anesthetized, the trachea was exposed and intubated, and 50 μL of beads suspension (10^5^ CFU) were injected before closing the incision with suture clips. Control mice were intratracheally inoculated with the same volume of empty beads suspension. After infection, mice were monitored daily for body weight, appetite and hair coat. Four days after infection, mice were euthanized by CO_2_ asphyxiation. Lung and spleen were collected, homogenized, and processed for microbiological analysis. Total CFU were the result of the addition of the CFU in lung homogenate and bronchoalveolar lavage fluid. Lung homogenates were centrifuged at 16,000 x g for 30 min at 4°C, and then stored at -80°C for cytokines and chemokines analysis. TNF, KC (CXCL1), and MIP-2 (CXCL2) concentrations were evaluated in the lung homogenate supernatants by DuoSet ELISA Development Systems (R&D Systems), according to the manufacturer’s instructions.

### Infection of *Galleria mellonella*

The *G*. *mellonella* model was used as previously described [48]. Briefly, larvae were infected by a 30G needle, with ~3000 CFU of *M*. *abscessus*, and kept at 37°C. Mortality was monitored daily. Where CFU count was needed, larvae were killed by freezing for 5 minutes at -20°C, de-contaminated in 70% ethanol, homogenized in 5 ml of PBS, and plated in dilutions on appropriate agar plates.

### Determination of MICs for ATCC_19977, ΔB11 and ΔB11+B11 strains, the clinical isolate LAH, and derivative and LAH+B11^MOP^

A luminescent mutant of ATCC_19977 was already created and described by us [48,97]. We created a similar luminescent mutant on a ∆B11 background, using the same integrating, kanamycin-resistance vector (pLux13). By cloning B11 with the native promoter into pLux13, we created a ΔB11+B11, luminescent mutant. 500 CFU of each were inoculated into 200 μl of 7H9 media, with serial dilutions of the designated antibiotics, in a luminescence-compatible 96-well plate. Bacteria were kept at 37°C, and growth was monitored daily by measurement of the luminescence by a Spectramax plate reader.

To complement the clinical strain LAH by B11, we first cloned B11 under the constitutive promoter MOP, so it is not dependent on the physiologic regulation of B11 in LAH, that appeared to be downregulated. MOP-B11 was then cloned into an integrating vector (pDB406), selected by both zeocin and kanamycin, to ensure selection in the highly resistant parent strain. pDB406 was electroporated into LAH to create LAH+B11^MOP^. For lack of additional effective selection markers, this mutant could not be made luminescent. To test for MIC, we therefore plated LAH and LAH+B11^MOP^ on LB/dextrose/glycerol plates supplemented by the designated concentration of antibiotics.

### Identification of mutations in clinical isolates

The genomes reported in [61] were analyzed. Fastq files were aligned to NC_010397.1 with bwa (91) and sequence variants identified by Samtools and Bamtools [98]. Read depth was verified with Artemis [99]. Genomes of isolates from Denmark were analyzed using a similar approach. Genome sequences of isolates from an additional 750 patients (S4 Table, [63–70] were analyzed by Snippy [100] to identify single-nucleotide polymorphisms and insertions/deletions (indels). Snippy uses BWA to align reads from many isolates to a reference genome and identifies variants among alignments using FreeBayes. The reference genome NC_010397.1 was used in all cases. Variants (S5 Table) were called only if the number of reads covering the site was greater than 10.

### Other statistical analyses and data visualizations

Graphpad Prism version 9.2.0 was used for most statistical analyses. Venn diagrams were made by BioVenn [101]. Hypergeometric tests were done at https://systems.crump.ucla.edu/hypergeometric/.

## Supporting information

S1 Fig Disruption of the sRNA B11 by a transposon and by targeted replacement. A. Schematic showing the position of transposon insertion at the M. abscessus B11 locus, and alignment of the surrounding sequence with the equivalent portion of the M. tuberculosis B11 locus. The promoter -10 sites are underlined. Three different 5’ ends were reported for M. tuberculosis B11, indicted with bold red font. B. Genomic context of B11 in three mycobacterial species. Not to scale. C. PCR reactions with primers binding 700 bp upstream and downstream of B11 were performed on WT and on the deletion candidate. The reaction yields a 1500 bp product in WT, and a 1900 bp product when B11 is replaced by the 500 bp zeocinR gene. D. Deletion of B11 caused extensive clumping during growth in liquid media without Tween. E. Predicted secondary structure of B11 (Vienna RNAfold). Boxed positions were mutated as follows in B11mutated shown in Fig 1A: G to A, C to U, and U to C.
(PDF)

S2 Fig LC-MS analysis of GPLs produced by the WT, B11 deletion mutant, and complemented mutant strain reveals similar relative abundance of diglycosylated and triglycosylated GPL species. Comparison of the relative abundance of diglycosylated (A) and triglycosylated GPL species (B) found in the M. abscessus WT, ΔB11, and ΔB11+B11 strains. LC-MS analysis revealed the presence of several GPL isomeric species containing 23–35 carbon atoms in their saturated acyl chains. The area under the peaks of the extracted ion chromatograms was used to calculate relative abundance in percentages (peak areas of multiple isomers were combined for a given acyl chain length). Insets represent the chemical structures of diglycosylated and triglycosylated GPLs.
(PDF)

S3 Fig A B11 deletion strain and a GPL biosynthesis mutant both release more protein into culture supernatants than smooth strains. A. Protein was extracted from cell pellets or from culture supernatants. Quantities from equivalent culture volumes were separated by SDS-PAGE and stained with Coomassie blue. The ∆B11 strain consistently released more protein into the culture supernatant than the WT or complemented strains. B. The same assay revealed that a rough strain with a transposon disruption of msp2 also released more protein into culture supernatants than the smooth WT parent. C. The indicated strains were transformed with a plasmid expressing mCherry (which lacks a secretion signal sequence) and their cell lysates and culture supernatants were probed by western blotting against mCherry. mCherry was exclusively cytoplasmic in the WT strain as expected, but present at detectable levels in the culture supernatants of the rough strains.
(PDF)

S4 Fig The genetic architecture of selected ESX-related genes repressed by B11. Coding sequences are shown in black and intergenic sequences are shown in red. The 50 nt sequence upstream of each start codon is shown. Start codons are bolded. Regions 6 nt or longer of complementarity to either loop of B11 are bolded and underlined. Shine-Dalgarno sequences in mycobacteria are typically located between -13 and -7 relative to the start codon (Shell et al 2015 and Martini et al 2019). Published transcription start sites (Miranda-CasoLuengo et al 2016) are indicated with bent arrows. Note that the TSS proximal to MAB_0630 had much lower read depth compared to the TSS upstream of MAB_0628, suggesting that the genes may be transcribed primarily as a polycistron. The sigM gene is likely transcribed as a leaderless mRNA; its annotated 5’ UTR is 6 nt, but the transcript begins with an in-frame GTG and we have previously shown that such transcripts are translated from their 5’ ends (Shell et al 2015). Both the annotated (downstream) and corrected (upstream) start codons are shown here. A sequence in the coding region that is complementary to 6 nt of B11 loop 1 or 6 nt of B11 loop 2 is bolded and underlined. Elements are not drawn to scale.
(PDF)

S5 Fig Expression of mCherry from a construct containing the MOP promoter and 5’ UTR is not affected by deletion of B11. A. Schematic of the MOP promoter+UTR reporter construct, and sequence of the 5’ UTR (start codon is bolded and underlined). B. mCherry fluorescence from the indicated strains containing the MOP promoter+UTR reporter construct, measured by flow cytometry. Data are mean and SD of biological triplicate cultures. Results are representative of two independent experiments. C. The complete sequence of the M. abscessus eccB4 5’ UTR used in Fig 5 (start codon is bolded and underlined). The sequence complementary to the B11 loops is indicated in bolded red.
(PDF)

S6 Fig B11 mutations found in clinical M. abscessus strains do not affect abundance when expressed ectopically in a B11 deletion strain. Total RNA from triplicate cultures of the indicated strains was transferred to a membrane and probed sequentially for B11 and 5S rRNA as a loading control.
(PDF)

S7 Fig PCA of RNAseq read counts. A. All three replicates of the five indicated strains were clustered based on read counts generated by FeatureCount. The circled replicates were eliminated from further analysis due to being outliers. Additionally, the eliminated WT replicate had a very low read count and the eliminated complemented replicate lacked detectable expression of B11. B. The remaining samples were clustered after removed of the samples indicated in A.
(PDF)

S1 Table Results from DESeq2 differential expression analysis, proteomic analysis, and identification of B11-complementary sequences in the ribosome-binding regions of genes with defined 5’ UTRs. (XLSX)

S2 Table Results from DESeq2 differential expression analysis and identification of B11-complementary sequences in the ribosome-binding regions of genes with defined 5’ UTRs, for GPL biosynthesis and transport genes only. (XLSX)

S3 Table Results from DESeq2 differential expression analysis and identification of B11-complementary sequences in the ribosome-binding regions of genes with defined 5’ UTRs, for genes differentially expressed in ΔB11, ΔB11+B11_del1_, and/or ΔB11+B11_ins1_ compared to ΔB11+B11. (XLSX)

S4 Table Publicly available *M*. *abscessus* genomes used in this work. (XLSX)

S5 Table Additional *M*. *abscessus* clinical isolates with mutations in B11. (XLSX)

S6 Table Sequences of primers and northern blot probes used in this work. (XLSX)

S7 Table Strains used in this work. (XLSX)
