# Supplementary material for: The small non-coding RNA B11 regulates multiple facets of Mycobacterium abscessus virulence
Source: PLoS Pathog. 2023 Aug 21;19(8):e1011575. doi: 10.1371/journal.ppat.1011575 (PMC10470900; doi:10.1371/journal.ppat.1011575)
Supplement: S1 Fig — A. Schematic showing the position of transposon insertion at the M. abscessus B11 locus, and alignment of the surrounding sequence with the equivalent portion of the M. tuberculosis B11 locus. The promoter -10 sites are underlined. Three different 5’ ends were reported for M. tuberculosis B11, indicted with bold red font. B. Genomic context of B11 in three mycobacterial species. Not to scale. C. PCR reactions with primers binding 700 bp upstream and downstream of B11 were performed on WT and on the deletion candidate. The reaction yields a 1500 bp product in WT, and a 1900 bp product when B11 is replaced by the 500 bp zeocinR gene. D. Deletion of B11 caused extensive clumping during growth in liquid media without Tween. E. Predicted secondary structure of B11 (Vienna RNAfold). Boxed positions were mutated as follows in B11mutated shown in Fig 1A: G to A, C to U, and U to C. (PDF) [file ppat.1011575.s001.pdf]

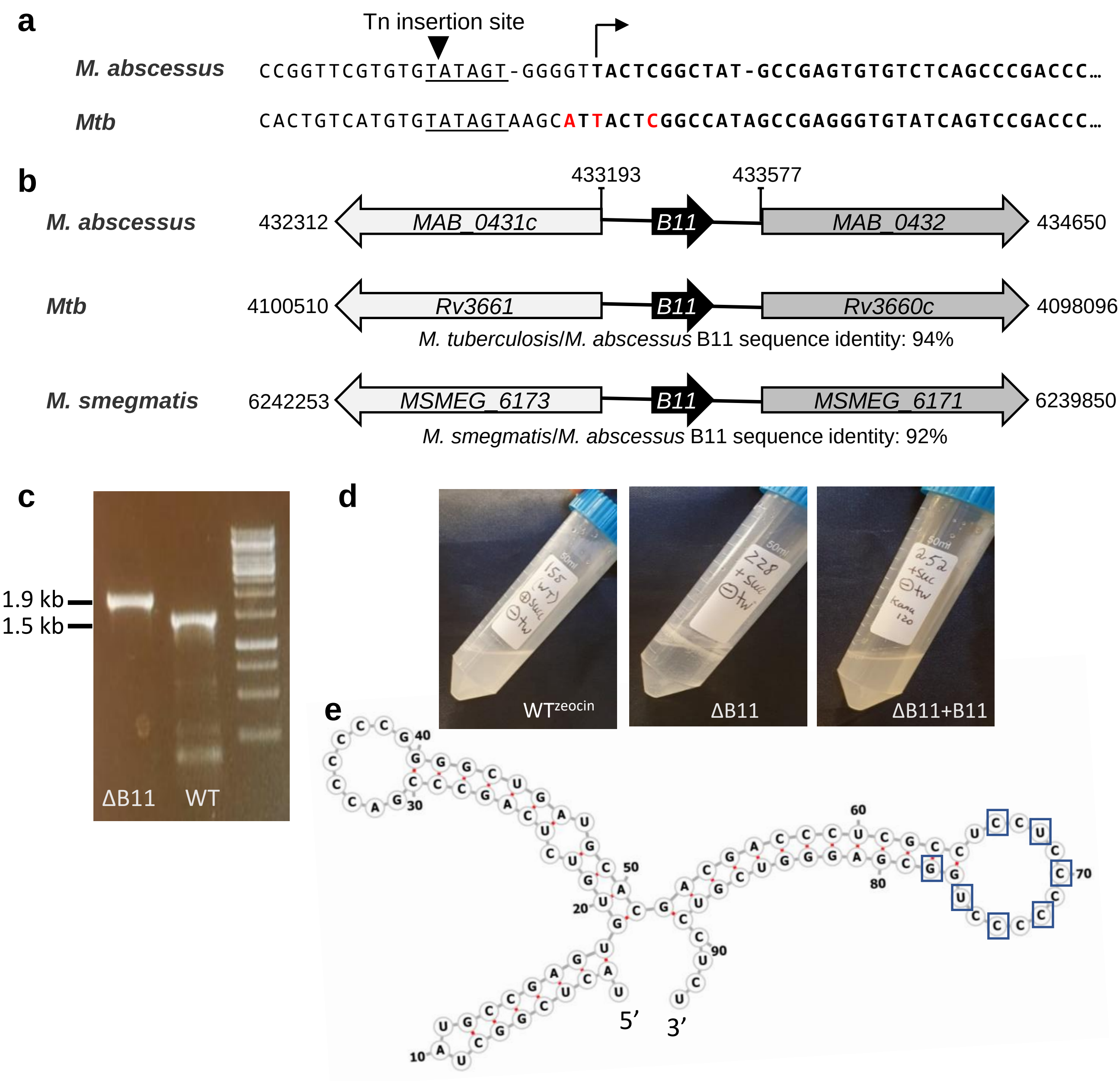

Figure S1. Disruption of the sRNA B11 by a transposon and by targeted replacement. A. Schematic showing the position of transposon insertion at the *M. abscessus* B11 locus, and alignment of the surrounding sequence with the equivalent portion of the *M. tuberculosis* B11 locus. The promoter -10 sites are underlined. Three different 5’ ends were reported for *M. tuberculosis* B11, indicted with bold red font. **B.** Genomic context of B11 in three mycobacterial species. Not to scale. **C.** PCR reactions with primers binding 700 bp upstream and downstream of B11 were performed on WT and on the deletion candidate. The reaction yields a 1500 bp product in WT, and a 1900 bp product when B11 is replaced by the 500 bp zeocin^R gene. **D.** Deletion of B11 caused extensive clumping during growth in liquid media without Tween. **E.** Predicted secondary structure of B11 (Vienna RNAfold). Boxed positions were mutated as follows in B11$_{mutated}$ shown in Fig 1a: G to A, C to U, and U to C.

Figure S1
