# Supplementary material for: The small non-coding RNA B11 regulates multiple facets of Mycobacterium abscessus virulence
Source: PLoS Pathog. 2023 Aug 21;19(8):e1011575. doi: 10.1371/journal.ppat.1011575 (PMC10470900; doi:10.1371/journal.ppat.1011575)
Supplement: S2 Fig — Comparison of the relative abundance of diglycosylated (A) and triglycosylated GPL species (B) found in the M. abscessus WT, ΔB11, and ΔB11+B11 strains. LC-MS analysis revealed the presence of several GPL isomeric species containing 23–35 carbon atoms in their saturated acyl chains. The area under the peaks of the extracted ion chromatograms was used to calculate relative abundance in percentages (peak areas of multiple isomers were combined for a given acyl chain length). Insets represent the chemical structures of diglycosylated and triglycosylated GPLs. (PDF) [file ppat.1011575.s002.pdf]

a

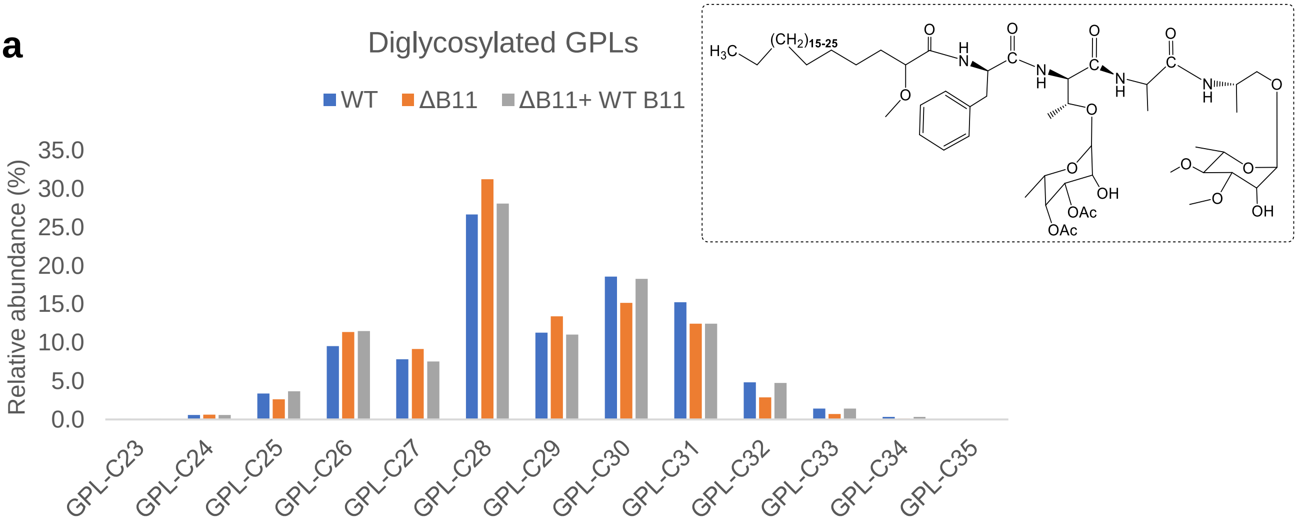

b

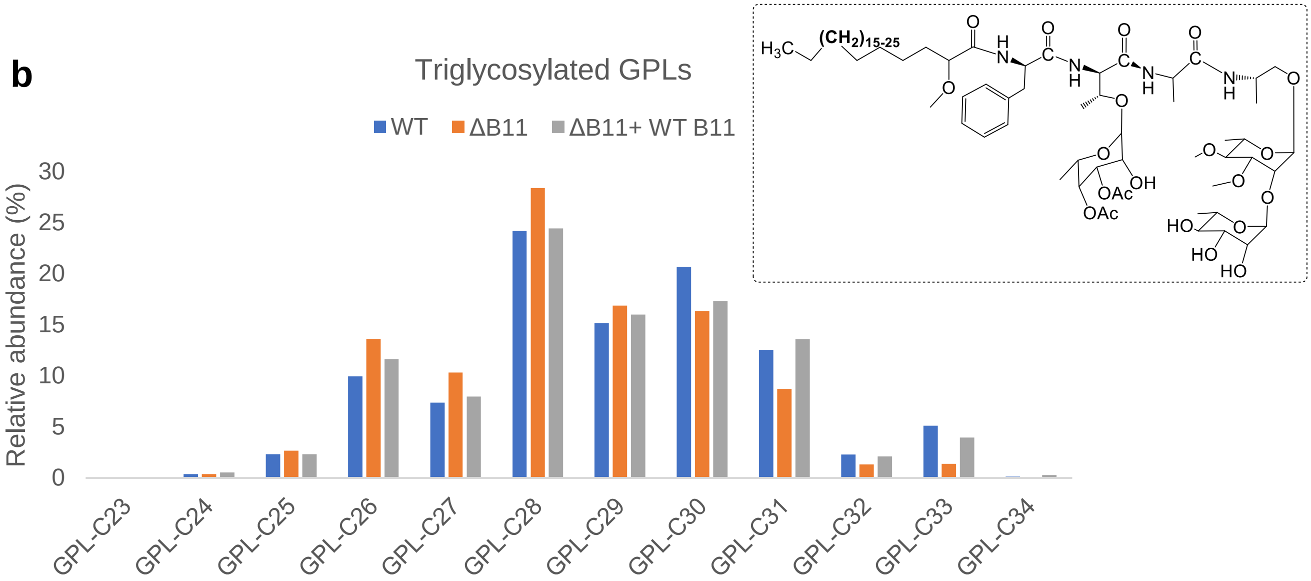

Figure S2. LC-MS analysis of GPLs produced by the WT, B11 deletion mutant, and complemented mutant strain reveals similar relative abundance of diglycosylated and triglycosylated GPL species. Comparison of the relative abundance of diglycosylated (A) and triglycosylated GPL species (B) found in the *M. abscessus* WT, ΔB11, and ΔB11+B11 strains. LC-MS analysis revealed the presence of several GPL isomeric species containing 23-35 carbon atoms in their saturated acyl chains. The area under the peaks of the extracted ion chromatograms was used to calculate relative abundance in percentages (peak areas of multiple isomers were combined for a given acyl chain length). Insets represent the chemical structures of diglycosylated and triglycosylated GPLs.

Figure S2
