# Supplementary material for: The small non-coding RNA B11 regulates multiple facets of Mycobacterium abscessus virulence
Source: PLoS Pathog. 2023 Aug 21;19(8):e1011575. doi: 10.1371/journal.ppat.1011575 (PMC10470900; doi:10.1371/journal.ppat.1011575)
Supplement: S3 Fig — A. Protein was extracted from cell pellets or from culture supernatants. Quantities from equivalent culture volumes were separated by SDS-PAGE and stained with Coomassie blue. The ∆B11 strain consistently released more protein into the culture supernatant than the WT or complemented strains. B. The same assay revealed that a rough strain with a transposon disruption of msp2 also released more protein into culture supernatants than the smooth WT parent. C. The indicated strains were transformed with a plasmid expressing mCherry (which lacks a secretion signal sequence) and their cell lysates and culture supernatants were probed by western blotting against mCherry. mCherry was exclusively cytoplasmic in the WT strain as expected, but present at detectable levels in the culture supernatants of the rough strains. (PDF) [file ppat.1011575.s003.pdf]

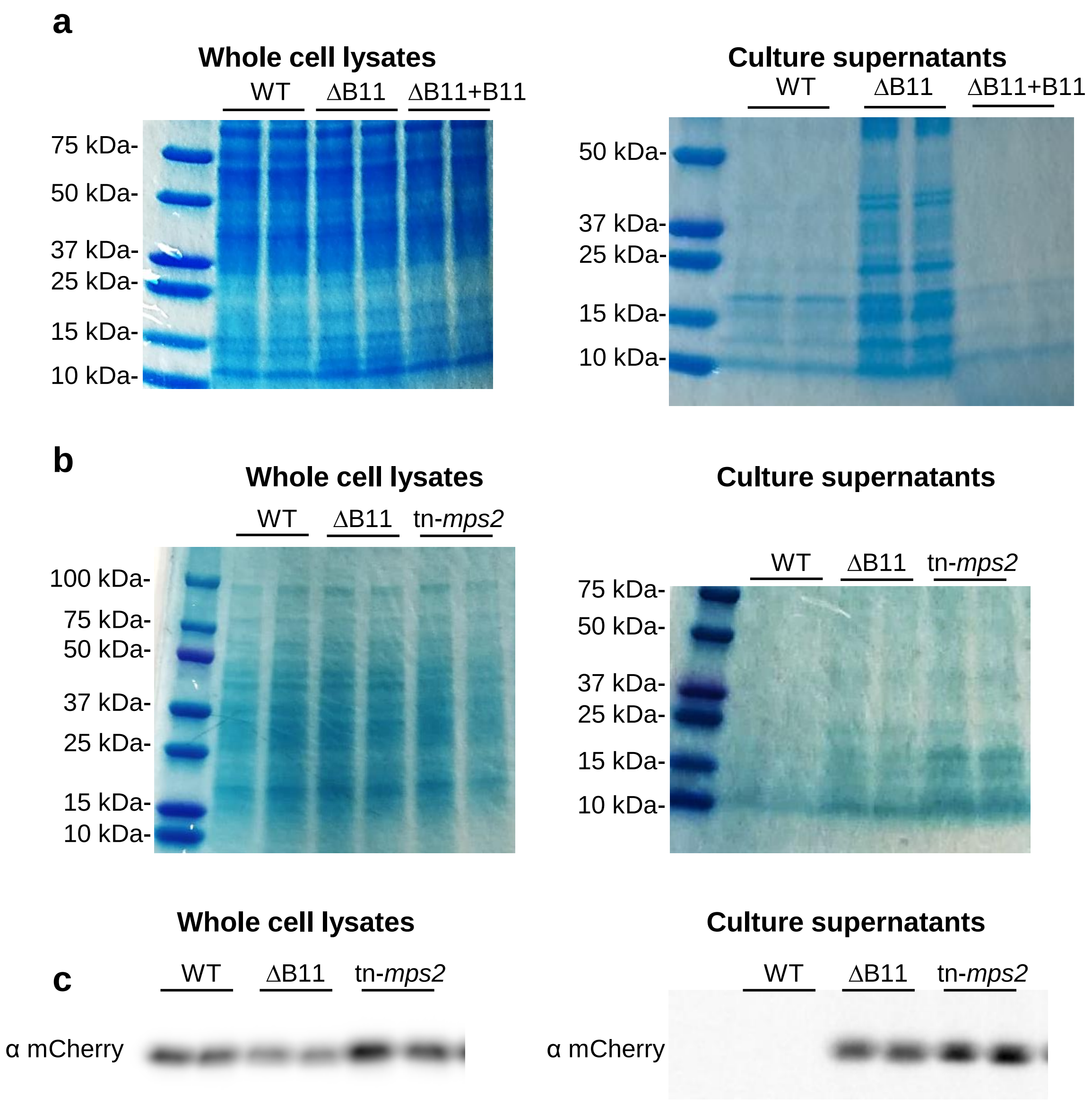

Figure S3. A B11 deletion strain and a GPL biosynthesis mutant both release more protein into culture supernatants than smooth strains. A. Protein was extracted from cell pellets or from culture supernatants. Quantities from equivalent culture volumes were separated by SDS-PAGE and stained with Coomassie blue. The ΔB11 strain consistently released more protein into the culture supernatant than the WT or complemented strains. **B.** The same assay revealed that a rough strain with a transposon disruption of *msp2* also released more protein into culture supernatants than the smooth WT parent. **C.** The indicated strains were transformed with a plasmid expressing mCherry (which lacks a secretion signal sequence) and their cell lysates and culture supernatants were probed by western blotting against mCherry. mCherry was exclusively cytoplasmic in the WT strain as expected, but present at detectable levels in the culture supernatants of the rough strains.

Figure S3
