# Supplementary material for: The small non-coding RNA B11 regulates multiple facets of Mycobacterium abscessus virulence
Source: PLoS Pathog. 2023 Aug 21;19(8):e1011575. doi: 10.1371/journal.ppat.1011575 (PMC10470900; doi:10.1371/journal.ppat.1011575)
Supplement: S4 Fig — Coding sequences are shown in black and intergenic sequences are shown in red. The 50 nt sequence upstream of each start codon is shown. Start codons are bolded. Regions 6 nt or longer of complementarity to either loop of B11 are bolded and underlined. Shine-Dalgarno sequences in mycobacteria are typically located between -13 and -7 relative to the start codon (Shell et al 2015 and Martini et al 2019). Published transcription start sites (Miranda-CasoLuengo et al 2016) are indicated with bent arrows. Note that the TSS proximal to MAB_0630 had much lower read depth compared to the TSS upstream of MAB_0628, suggesting that the genes may be transcribed primarily as a polycistron. The sigM gene is likely transcribed as a leaderless mRNA; its annotated 5’ UTR is 6 nt, but the transcript begins with an in-frame GTG and we have previously shown that such transcripts are translated from their 5’ ends (Shell et al 2015). Both the annotated (downstream) and corrected (upstream) start codons are shown here. A sequence in the coding region that is complementary to 6 nt of B11 loop 1 or 6 nt of B11 loop 2 is bolded and underlined. Elements are not drawn to scale. (PDF) [file ppat.1011575.s004.pdf]

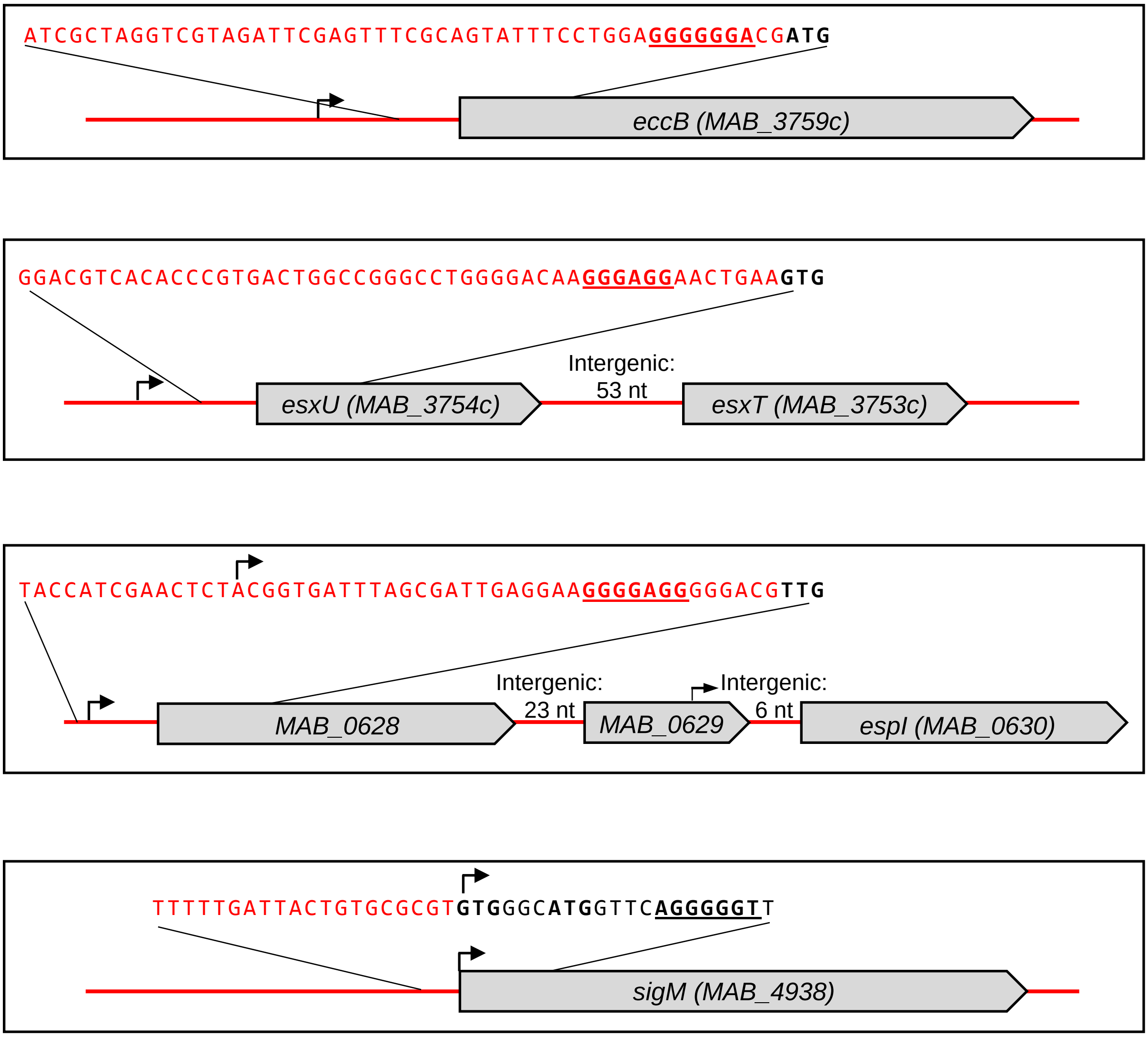

Figure S4. The genetic architecture of selected ESX-related genes repressed by B11. Coding sequences are shown in black and intergenic sequences are shown in red. The 50 nt sequence upstream of each start codon is shown. Start codons are bolded. Regions 6 nt or longer of complementarity to either loop of B11 are bolded and underlined. Shine-Dalgarno sequences in mycobacteria are typically located between -13 and -7 relative to the start codon (Shell et al 2015 and Martini et al 2019). Published transcription start sites (Miranda-CasoLuengo et al 2016) are indicated with bent arrows. Note that the TSS proximal to *MAB_0630* had much lower read depth compared to the TSS upstream of *MAB_0628*, suggesting that the genes may be transcribed primarily as a polycistron. The *sigM* gene is likely transcribed as a leaderless mRNA; its annotated 5' UTR is 6 nt, but the transcript begins with an in-frame GTG and we have previously shown that such transcripts are translated from their 5' ends (Shell et al 2015). Both the annotated (downstream) and corrected (upstream) start codons are shown here. A sequence in the coding region that is complementary to 6 nt of B11 loop 1 or 6 nt of B11 loop 2 is bolded and underlined. Elements are not drawn to scale.

Figure S4
