# Supplementary material for: The small non-coding RNA B11 regulates multiple facets of Mycobacterium abscessus virulence
Source: PLoS Pathog. 2023 Aug 21;19(8):e1011575. doi: 10.1371/journal.ppat.1011575 (PMC10470900; doi:10.1371/journal.ppat.1011575)
Supplement: S5 Fig — A. Schematic of the MOP promoter+UTR reporter construct, and sequence of the 5’ UTR (start codon is bolded and underlined). B. mCherry fluorescence from the indicated strains containing the MOP promoter+UTR reporter construct, measured by flow cytometry. Data are mean and SD of biological triplicate cultures. Results are representative of two independent experiments. C. The complete sequence of the M. abscessus eccB4 5’ UTR used in Fig 5 (start codon is bolded and underlined). The sequence complementary to the B11 loops is indicated in bolded red. (PDF) [file ppat.1011575.s005.pdf]

**a**

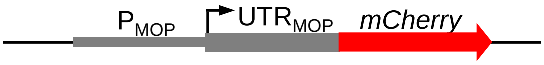


**UTR$_{MOP}$:**

5’AATTGTGAGCGCTCACAATTCGGATCCAGCTGCAGAATTCGAAGGAGATATACAT**<u>ATG</u>** 3’

**b**

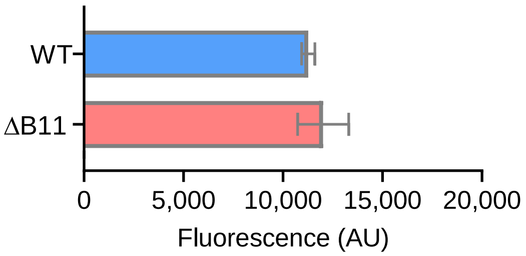


**c**

**UTR*eccB4*:**

5’GGAGGCATCGCTAGGTCGTAGATTCGAGTTTCGCAGTATTTCCTGGA**GGGGGGA**CG**<u>ATG</u>** 3’

**Figure S5. Expression of mCherry from a construct containing the MOP promoter and 5’ UTR is not affected by deletion of B11. A.** Schematic of the MOP promoter+UTR reporter construct, and sequence of the 5’ UTR (start codon is bolded and underlined). **B.** mCherry fluorescence from the indicated strains containing the MOP promoter+UTR reporter construct, measured by flow cytometry. Data are mean and SD of biological triplicate cultures. Results are representative of two independent experiments. **C.** The complete sequence of the *M. abscessus eccB4* 5’ UTR used in Fig. 5 (start codon is bolded and underlined). The sequence complementary to the B11 loops is indicated in bolded red.

Figure S5
