# Supplementary material for: The small non-coding RNA B11 regulates multiple facets of Mycobacterium abscessus virulence
Source: PLoS Pathog. 2023 Aug 21;19(8):e1011575. doi: 10.1371/journal.ppat.1011575 (PMC10470900; doi:10.1371/journal.ppat.1011575)
Supplement: S6 Fig — Total RNA from triplicate cultures of the indicated strains was transferred to a membrane and probed sequentially for B11 and 5S rRNA as a loading control. (PDF) [file ppat.1011575.s006.pdf]

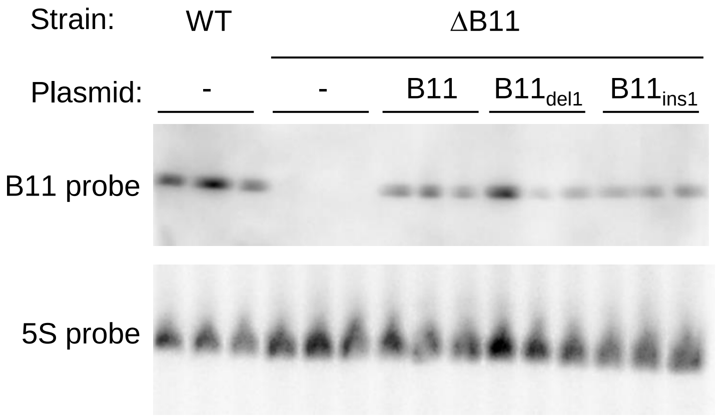

Figure S6. B11 mutations found in clinical *M. abscessus* strains do not affect abundance when expressed ectopically in a B11 deletion strain. Total RNA from triplicate cultures of the indicated strains was transferred to a membrane and probed sequentially for B11 and 5S rRNA as a loading control.

Figure S6
