# Supplementary material for: The small non-coding RNA B11 regulates multiple facets of Mycobacterium abscessus virulence
Source: PLoS Pathog. 2023 Aug 21;19(8):e1011575. doi: 10.1371/journal.ppat.1011575 (PMC10470900; doi:10.1371/journal.ppat.1011575)
Supplement: S7 Fig — A. All three replicates of the five indicated strains were clustered based on read counts generated by FeatureCount. The circled replicates were eliminated from further analysis due to being outliers. Additionally, the eliminated WT replicate had a very low read count and the eliminated complemented replicate lacked detectable expression of B11. B. The remaining samples were clustered after removed of the samples indicated in A. (PDF) [file ppat.1011575.s007.pdf]

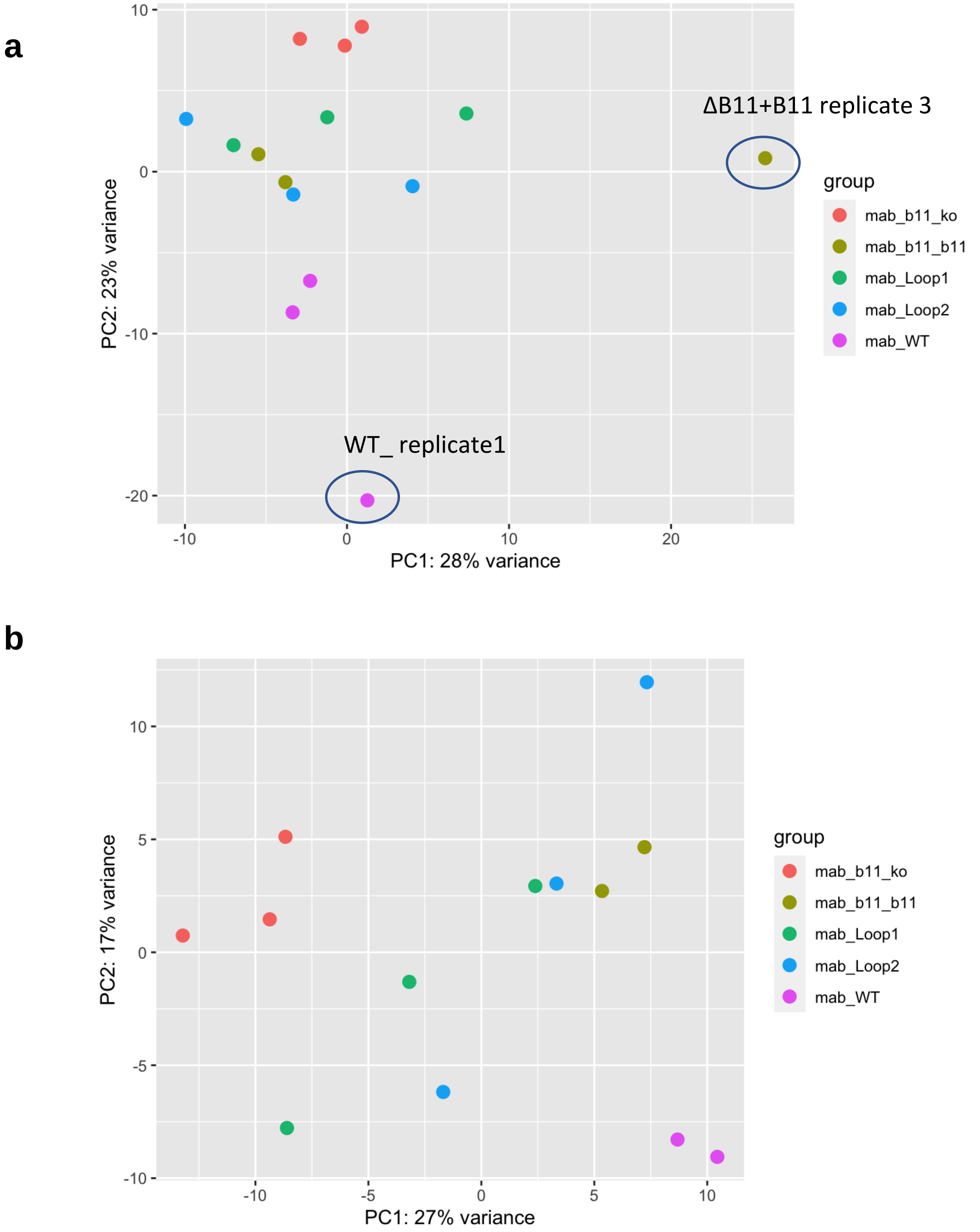

Figure S7. PCA of RNAseq read counts. A. All three replicates of the five indicated strains were clustered based on read counts generated by FeatureCount. The circled replicates were eliminated from further analysis due to being outliers. Additionally, the eliminated WT replicate had a very low read count and the eliminated complemented replicate lacked detectable expression of B11. **B.** The remaining samples were clustered after removed of the samples indicated in A.

Figure S7
